# Pathogenic G*α*o Mutants Drive Dominant GPCR Coupling in *GNAO1* Encephalopathies

**DOI:** 10.1096/fj.202503265R

**Published:** 2025-12-29

**Authors:** Yonika A. Larasati, Camille Rabesahala de Meritens, Miriam Stoeber, Vladimir L. Katanaev, Gonzalo P. Solis

**Affiliations:** ^1^ Translational Research Center in Oncohaematology, Department of Cell Physiology and Metabolism, Faculty of Medicine University of Geneva Geneva Switzerland; ^2^ Department of Cell Physiology and Metabolism, Faculty of Medicine University of Geneva Geneva Switzerland; ^3^ Translational Oncology Research Center, Qatar Biomedical Research Institute (QBRI), College of Health and Life Sciences Hamad Bin Khalifa University (HBKU), Qatar Foundation Doha Qatar

**Keywords:** bimolecular fluorescence complementation (BiFC), dystonia, epilepsy, G protein‐coupled receptors (GPCRs), GNAO1, G*α*o, heterotrimeric G proteins, movement disorders, neurodevelopmental disorders

## Abstract

Heterozygous mutations in *GNAO1*, which encodes the G*α*o subunit of heterotrimeric G proteins, cause a spectrum of neurodevelopmental disorders ranging from early‐onset epileptic encephalopathy to dystonia. Although the mechanisms underlying disease dominance remain incompletely understood, some functional disruptions in G*α*o mutants have been described. Intriguingly, several G*α*o variants have been independently reported to dominantly engage G protein‐coupled receptors (GPCRs) or to sequester G*β*γ—two seemingly incompatible mechanisms inferred from distinct, indirect biosensor assays. To clarify this apparent contradiction, we developed a split‐YFP‐based bimolecular fluorescence complementation (BiFC) assay to directly visualize receptor‐G_o_ protein complexes at the plasma membrane. Using this system, we found that severe G*α*o variants fail to disengage from activated Gi/o‐coupled GPCRs, thereby preventing downstream receptor phosphorylation and endocytosis. By contrast, milder dystonia‐linked mutants showed near‐normal receptor internalization and only minor phosphorylation defects. These findings establish dominant GPCR coupling as a molecular hallmark of severe *GNAO1* encephalopathies and point to split‐YFP BiFC as a robust platform for probing mutant G protein behavior in genetic disease.

## Graphical abstract

Mutations in *GNAO1*, the gene encoding G*α*o, are associated with a wide range of neurodevelopmental disorders. In this study, we show that G*α*o variants linked to the most severe patient phenotypes fail to disengage from activated Gi/o‐coupled GPCRs, thereby blocking GRK‐mediated receptor phosphorylation and internalization. These results identify dominant GPCR coupling by G*α*o mutants as a defining molecular feature of severe *GNAO1* encephalopathies.

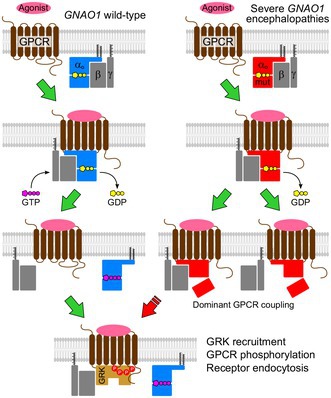

## 1 Introduction

G protein‐coupled receptors (GPCRs) represent the largest and most diverse family of membrane receptors in metazoans [1, 2]. From neurotransmission and hormonal signaling to sensory perception and immune responses, GPCRs are essential for proper animal development and physiology [3, 4]. Signal transduction by GPCRs is mediated by heterotrimeric G proteins, composed of three subunits: *α*, *β*, and γ, with the G*α* subunit conferring receptor specificity [5, 6]. In the inactive state, G*α* is bound to GDP and associates tightly with the G*β*γ dimer. Upon ligand binding, the GPCR undergoes a conformational change that catalyzes the exchange of GDP for GTP on G*α*. This process activates the heterotrimeric G protein, causing G*α*‐GTP and G*β*γ to dissociate and signal independently to downstream effectors. The signal is terminated when G*α* hydrolyzes GTP to GDP through its intrinsic GTPase activity and associates with G*β*γ, reforming the heterotrimer [7, 8]. Given their central role in GPCR signaling, it is perhaps unsurprising that mutations affecting heterotrimeric G protein subunits have been implicated in a variety of human diseases.

Historically, gain‐of‐function mutations in genes encoding G*α* subunits have drawn most of the attention due to their role as drivers in various cancers [9, 10]. These mutations produce constitutively active G*α* proteins, resulting in persistent stimulation of oncogenic signaling pathways. More recently, however, a growing number of rare genetic disorders have been attributed to alternative mutations in G*α* subunits [11, 12]. Among these, mutations in *GNAO1*, the gene encoding for G*α*o, have been implicated in a broad spectrum of neurodevelopmental disorders [13, 14, 15]. The most severe conditions were originally classified as Developmental and Epileptic Encephalopathy 17 (DEE17; OMIM #615473) and Neurodevelopmental Disorder with Involuntary Movements (NEDIM; OMIM #617493) [16, 17]. In recent years, *GNAO1* mutations have also been associated with milder conditions, including autism and intellectual disability, parkinsonism, and dystonia [18, 19, 20]. More than 250 patients have been reported worldwide, the vast majority carrying missense mutations, with only a few cases of deletions, frameshifts, duplications, short indels, and splice‐site mutations described [15]. Treatment responses among *GNAO1* patients are highly variable and typically poor, particularly in cases involving epilepsy and/or movement disorders. This underscores the urgent need for more effective therapies for these severe conditions.

At the molecular level, G*α*o mutants associated with severe phenotypes display a set of interconnected pathogenic mechanisms, including severely impaired guanine nucleotide handling, misfolding in the GTP‐bound active state, and neomorphic binding to Ric8A/B chaperones, all of which converge to perturb the broader GPCR signaling network [21, 22, 23, 24, 25, 26]. By contrast, variants on the milder end of the *GNAO1*‐related disorder spectrum exhibited only mild biochemical defects, with some mutants lacking the neomorphic property entirely, while others retained trapping of Ric8A but not Ric8B [19, 20, 23, 27]. Furthermore, the most severe DEE17 G*α*o variants tend to lose plasma membrane (PM) localization and G*β*γ binding, whereas mutants linked to NEDIM or milder phenotypes retain near‐normal association with both PM and G*β*γ [21, 22, 23].

Another emerging feature of several pathogenic *GNAO1* mutations is their apparent dominant coupling to GPCRs. A study using a bioluminescence resonance energy transfer (BRET)‐based assay to measure free G*β*γ during GPCR stimulation showed that clinically severe G*α*o variants partially inhibit activation of the wild‐type protein, acting in a dominant‐negative (DN) manner [21]. These G*α*o mutants displayed poor disengagement from G*β*γ despite normal (or even enhanced) coupling to activated GPCRs—the latter determined indirectly by a split‐NanoLuc assay measuring G*β*γ proximity to the receptor. Based on this, the authors proposed that the DN effect arises from nonproductive interactions between the mutant G*α*o and GPCRs. Along the same lines, we found that many clinically severe G*α*o mutants exhibit normal or even enhanced GPCR coupling despite poor binding to G*β*γ, using a different BRET‐based assay that directly monitors GPCR engagement by G*α* [23]. This dominant coupling mechanism was recently confirmed for the epileptic G*α*o K46E variant, which forms a nonproductive complex with both G*β*γ and the activated GPCR, as revealed by cryo‐electron microscopy [28]. By contrast, an earlier study using yet another BRET‐based assay that directly measures G*β*γ disengagement from G*α* described an alternative DN mechanism for some of the same G*α*o mutants: sequestration of G*β*γ, due to impaired adoption of the active conformation required for their dissociation, without sequestering the GPCR [22].

Thus, dominant GPCR coupling by pathogenic G*α*o mutants can currently only be inferred using a combination of optical biosensors, leading to apparent mechanistic contradictions. To resolve this, we developed a novel assay to directly visualize the dominant GPCR coupling behavior of G*α*o variants. We employed bimolecular fluorescence complementation (BiFC), a method based on the structural reassembly of two non‐fluorescent N‐ and C‐terminal fragments of a fluorescent protein into a functional fluorophore when both fragments are in close proximity [29]. Using the split‐YFP‐based BiFC approach, we show that *GNAO1* mutations associated with the most severe phenotypes result in dominant GPCR coupling by the mutant G*α*o, likely caused by a defective receptor‐G*α* uncoupling.

## 2 Material and Methods

### 2.1 Antibodies and Reagents

Primary antibodies (Abs) for immunofluorescence (IF) and Western blots (WBs): monoclonal Abs (mAbs) anti‐G*α*o (clone A2, sc‐13 532, RRID:AB_2111645; IF: 1/50) and anti‐G*α*o (clone E1, sc‐393 874, RRID:AB_2941093; WB: 1/250) were from Santa Cruz Biotechnology (Dallas, TX, USA); mAb anti‐HA‐tag (clone 3F10, 11 867 423 001, RRID:AB_390918; IF: 1/500) was from Roche (Basel, Switzerland). Polyclonal antibodies (pAbs) anti‐M_2_R (7TM0014N; WB: 1/1000) and anti‐pT307/pS309 (7TM0014D; IF: 1/500, WB: 1/1000) were from 7TM Antibodies (Jena, Thuringia, Germany); pAbs anti‐GFP (PABG1, RRID:AB_2749857; IF: 1/1000) and anti‐GFP (50430–2‐AP, RRID:AB_11042881; WB: 1/5000) were from Proteintech (Rosemont, IL, USA).

All secondary Abs for immunofluorescence (IF) and Western blots (WBs) were from Jackson ImmunoResearch (West Grove, PA, USA): anti‐Mouse Cy5‐conjugated (715–175‐151, RRID:AB_2340820; IF: 1/500), anti‐Rabbit Cy3‐conjugated (711–165‐152, RRID:AB_2307443; IF: 1/500), anti‐Rat Cy3‐conjugated (112–165‐143, RRID:AB_2338250; IF: 1/500), anti‐mouse HRP‐conjugated (115–035‐146, RRID:AB_2307392; WB: 1/5000), and anti‐rabbit HRP‐conjugated (111–035‐144, RRID:AB_2307391; WB: 1/5000).

Acetylcholine (A2661), isoproterenol (I6504), and DAPI (32670) were from Sigma‐Aldrich (Burlington, MA, USA), and EGF‐Rhodamine (E3481) was from Thermo Fisher Scientific (Waltham, MA, USA). Fentanyl (CAS no. 1443‐54‐5) was obtained from the University Hospital of Geneva with Département de la sécurité, de la population et de la santé (DSPS) authorization from the Canton of Geneva.

### 2.2 Plasmids and Molecular Cloning

Plasmids encoding for the untagged G*α*o wild‐type and pathogenic mutants were published earlier [23, 27, 30]. Untagged G*α*o ins4A was kindly provided by Nevin A. Lambert (Augusta University, GA, USA). To generate the mRFP internal tagging of G*α*o (at Gly92; G*α*o‐mRFP), the mRFP sequence was amplified by PCR from pmRFP‐C1 [30] using the oligonucleotide primers For: 5′–GTCGCCGGGCCCGCCTCCTCCGAGGAC–3′ and Rev: 5′–TTTAAAGCAAGTAAAACCTC–3′. The PCR product was cloned in‐frame into the PspOMI/BsrGI sites of G*α*o‐GFP [31]. The sequences for the split‐YFP fragments YN (aa 1–158) and YC (aa 159–239) containing a flexible 10‐amino acid (GGGGSGGGGS) linker were kindly provided by Daniel F. Legler (Institute of Cell Biology and Immunology Thurgau, Switzerland). To produce the G*α*o‐YC constructs (internal YC insertion at Gly92), the YC sequence was amplified by PCR using the primers For: 5′–GGATCCACCGGTCCTCACCGGGCCCGGTGGCGGTGGCTCTGG–3′ and Rev: 5′–AGATCTGAGTCCGGACTTGTACACGGACCC–3′. The resulting product was cloned in‐frame into the PspOMI/BsrGI sites of G*α*o‐GFP wild‐type and mutants [23, 27]. The G*α*o‐YC for ins4A was obtained by replacing the AgeI/PspOMI fragment in G*α*o‐YC wild‐type with the AgeI/NotI fragment from untagged G*α*o ins4A. The GFP‐M_2_R construct was generated by cloning in‐frame the fragment blunted‐EcoRI/XhoI from the untagged M_2_R plasmid (MAR0200000; cDNA Resource Center, Bloomsburg, PA, USA) into the Eco53KI/SalI sites of the PrP‐leader‐GFP plasmid [32]. To produce the M_2_R‐YN construct, we first PCR amplified the YN sequence with the primers For: 5′–AGCCCGGACCGGTCCTCACCATGGATGGTGGCGGTGGCTC–3′ and Rev: 5′–TTTAGGTGGCGGCCGCGAATAGGACCCTCTAGATTAC–3′. The PCR product was digested with AgeI/NotI and ligated in‐frame into the AgeI/NotI sites of M_2_R‐NLuc [23]. The sequence for a signal peptide‐HA tag was generated by the primers For: 5′–TCGACCACCATGAAGACGATCATCGCCCTGAGCTACATCTTCTGCCTGGTATTCGCCTACCCATACGATGTTCCTGACTATGCGG–3′ and Rev: 5′–AATTCCGCATAGTCAGGAACATCGTATGGGTAGGCGAATACCAGGCAGAAGATGTAGCTCAGGGCGATGATCGTCTTCATGGTGG–3′ and cloned in‐frame into the XhoI/EcoRI sites upstream of the M_2_R. For the M_2_R‐YC plasmid, the YC fragment was amplified by PCR using the primers For: 5′–ACTATGAACCGGTACTCACCATGGATGGTGGCGGTGGCTC–3′ and Rev: 5′–GCAACTAGAAGGCACAGTCGAGG–3′, and cloned in‐frame into the AgeI/NotI sites of the final M_2_R‐YN. The *β*_2_AR‐YN plasmid was obtained by cutting the NheI/AgeI coding fragment from *β*_2_AR‐mCFP (plasmid 38260; Addgene, Watertown, MA, USA) and insertion into the same sites of M_2_R‐YN. The MOR‐YN construct was produced by cutting the XhoI/XmaI coding sequence from the MOR‐GFP plasmid [33], and ligation into the XhoI/AgeI sites of M_2_R‐YN. The T2A‐based bicistronic G*β*3‐YN‐Gγ3 construct—referred to as YN‐Gγ3 for simplicity—was generated through a two‐step cloning. First, the single YN‐Gγ3 plasmid was cloned by PCR amplification of the YN sequence using the primers For: 5′–CTATAGGACCGGTCAAGACCATGGTGAGCAAGGGCGAGGA–3′ and Rev: 5′–TGCTCGAGTGTACACGGACCCACCACCTCCAGAGC–3′, and the YN fragment was cloned in‐frame into the AgeI/BsrGI sites of GFP‐Gγ3 [30]. Then, a T2A‐based bicistronic G*β*3‐cpVenus‐Gγ9 plasmid was created by cutting the NheI/SalI insert from Go1‐CASE [34] (provided by Gunnar Schulte; Karolinska Institutet, Sweden) and ligation of the fragment into the same sites of pEGFP‐C1 (Clontech, San Jose, CA, USA). The final YN‐Gγ3 construct was generated by cutting the YN‐Gγ3 coding sequence with AfeI/PspOMI, and ligation of this fragment into the blunted‐AgeI/PspOMI of G*β*3‐cpVenus‐Gγ9. A T2A‐based bicistronic untagged G*β*3‐Gγ9 plasmid (referred to as G*β*3γ9) was generated by cutting out the cpVenus sequence from G*β*3‐cpVenus‐Gγ9 with AgeI/BspEI, and religation to produce an in‐frame G*β*3‐T2A‐Gγ9 construct.

### 2.3 Cell Lines and Culture Conditions

Human HEK293T (CRL‐3216, RRID:CVCL_0063; ATCC, Manassas, VA, USA) cells were grown in DMEM, supplemented with 10% FCS, 2 mM L‐glutamine, and 1% penicillin–streptomycin (all from Thermo Fisher Scientific, Waltham, MA, USA) at 37°C and 5% CO_2_. All vector transfections were carried out with X‐tremeGENE HP (XTGHP‐RO; Roche, Basel, Switzerland) according to the manufacturer's instructions.

### 2.4 Split‐YFP‐Based BiFC Assay and Microscopy

For the split‐YFP assay between GPCRs and G*α*o, HEK293T cells were co‐transfected with GPCR‐YN constructs (M_2_R, MOR, or *β*_2_AR), G*α*o‐YC (wild‐type, pathogenic mutants, or the control ins4A), and G*β*3γ9 at a plasmid ratio of 2.5:2.5:1. For the split‐YFP assay between M_2_R and Gγ3, cells were co‐transfected with M_2_R‐YC, YN‐Gγ3, and untagged G*α*o variants or empty plasmid at the same ratio. After 6 h, cells were trypsinized, seeded onto poly‐L‐lysine‐coated coverslips, and cultured—or co‐cultured in the case of the M_2_R‐Gγ3 split‐YFP assay—for an additional 15 h in complete DMEM.

For endocytosis analysis of GPCR‐YFP‐G*α*o complexes, cells were stimulated for 10, 20, and/or 30 min with 100 μM acetylcholine (ACh) for M_2_R, 1 μM fentanyl for MOR, or 10 μM isoproterenol for *β*_2_AR. To determine M_2_R phosphorylation, cells were stimulated with 100 μM ACh for 7 min. To evaluate EGF uptake, cells were treated with 10 ng/mL EGF‐Rhodamine for 10 min as previously described [35].

After treatments, cells were fixed with 4% PFA in PBS for 20 min and permeabilized with ice‐cold 0.1% Triton X‐100 in PBS for 1 min—this step was omitted under non‐permeabilizing conditions. Cells were then blocked for 1 h in PBS supplemented with 1% BSA (810 533; Millipore, Burlington, MA, USA), incubated with primary antibodies in blocking buffer for 2 h at room temperature (RT), washed, and subsequently incubated with fluorescently labeled secondary antibodies and DAPI in blocking buffer for 2 h at RT. Coverslips were mounted with VECTASHIELD (H‐1700; Vector Laboratories, Newark, CA, USA) on microscope slides. For immunostaining against phosphorylated M_2_R, all steps were performed in TBS instead of PBS.

Samples were recorded using a Plan‐Apochromat 63×/1.4 oil objective on an LSM800 confocal microscope with ZEN 3.7 software (all from Zeiss, Oberkochen, Baden‐Württemberg, Germany). When quantification was required, all images were acquired using identical confocal settings across all channels. Mean fluorescence intensities, cell area, and vesicle‐like structure counts were determined from confocal images either manually or semi‐automatically using in‐house scripts, all with ImageJ v1.54p (RRID:SCR_002285; National Institutes of Health).

### 2.5 PM Targeting of GFP‐M_2_R

HEK293T cells were co‐transfected with GFP‐M_2_R, untagged G*α*o variants (wild‐type, pathogenic mutants, or the control ins4A), and G*β*3γ9 at a plasmid ratio of 2.5:2.5:1. Samples were immunostained under non‐permeabilizing conditions, and image acquisition and quantification were performed as described above.

### 2.6 Immunoprecipitation

The recombinant GST‐tagged nanobody against GFP was expressed in *Escherichia coli* Rosettagami (71 351; Novagen, Madison, WI, USA) and purified using Glutathione Sepharose 4B beads according to the manufacturer's instructions. Protein purity was confirmed by SDS‐PAGE and Coomassie blue staining.

HEK293T cells were co‐transfected with the split‐YFP constructs for M_2_R, G*α*o, and G*β*3γ9, or with GFP‐M_2_R, untagged G*α*o variants, and G*β*3γ9, using the same plasmid ratio described above. After 24 h of transfection, cells were stimulated with 100 μM ACh for 5 min, then immediately resuspended in ice‐cold TBS‐lysis buffer (20 mM Tris–HCl, pH 7.5, 150 mM NaCl, 1% Triton X‐100, 10% glycerol) supplemented with protease (04693132001) and phosphatase (04906837001) inhibitor cocktails from Roche (Basel, Switzerland). Lysates were passed 15 times through a 25‐G needle and cleared by centrifugation at 15000xg for 15 min at 4°C. Supernatants were incubated with 2 μg of purified anti‐GFP nanobody for 30 min on ice, followed by addition of 20 μL of Glutathione Sepharose 4B beads (17 075 601; Cytiva, Marlborough, MA, USA) and rotation for 3 h at 4°C.

Beads were washed repeatedly with TBS‐lysis buffer and resuspended in Laemmli sample buffer. Inputs and beads were heated at 50°C for 30 min and analyzed by SDS‐PAGE Western blotting using Abs against phospho‐M_2_R (pT307/pS309), total M_2_R, G*α*o, and GFP. HRP‐conjugated secondary antibodies were used for detection, with all antibody incubations performed in TBS containing 1% BSA. Signal was visualized using enhanced chemiluminescence (ECL) on a Fusion FX6 Edge system (Vilber). Quantification of all WBs was performed using ImageJ v1.54p. Final image preparation was done in EvolutionCapt v18.11 (RRID:SCR_023580; Vilber, Collégien, Île‐de‐France, France) and CorelDRAW 2020 (RRID:SCR_014235; Corel Corporation, Ottawa, ON, Canada).

### 2.7 Statistics

Data were analyzed using GraphPad Prism (version 10.5.0; GraphPad Software, San Diego, CA, USA). Box‐and‐whisker plots indicate the median (middle line), the 25th and 75th percentiles (box), and the lowest and highest values (whiskers). Bar plots represent mean ± SEM. Multiple comparisons were assessed by one‐way ANOVA followed by Dunnett's or Šídák's multiple comparisons tests, as indicated. A *P* value < 0.05 was considered statistically significant. All *P* values are reported in the figures and legends.

## 3 Results

### 3.1 Dominant GPCR Coupling by Pathogenic G*α*o Mutants Blocks Receptor Endocytosis

To determine whether pathogenic G*α*o mutants indeed engage in dominant coupling with GPCRs, we sought to develop a single assay capable of visualizing this process. However, microscopic visualization of plasma membrane (PM)‐localized GPCRs can be challenging, as exogenous expression often leads to their accumulation in the endoplasmic reticulum and Golgi apparatus due to saturation of the cellular folding and trafficking machinery [36]. Consistent with this, expression of an N‐terminal GFP fusion of the M_2_ muscarinic acetylcholine receptor (GFP‐M_2_R) in HEK293T cells results in prominent intracellular retention (Figure S1A). We selected M_2_R for this study because it is a prototypical Gi/o‐coupled GPCR [37] and because we previously observed that severe G*α*o mutants exhibit enhanced coupling to this receptor [23], consistent with dominant GPCR engagement.

To overcome this limitation, we employed a split‐YFP‐based BiFC assay (hereafter split‐YFP), previously used to visualize the PM interaction between the chemokine GPCR CCR7 and the Src tyrosine kinase [38]. Like Src, G*α* subunits associate with the membrane via lipid modifications [39, 40], which target G*α*o to the PM and Golgi in HEK293T cells (Figure S1B) [30]. Based on these observations, we reasoned that a split‐YFP assay combining M_2_R and G*α*o would enable selective visualization of the PM‐localized receptor pool. We further speculated that the irreversible nature of the split‐YFP complementation would bias M_2_R toward coupling with G*α*o over endogenous G*α* subunits, thereby enhancing both the specificity and sensitivity of the assay.

**FIGURE 1:**
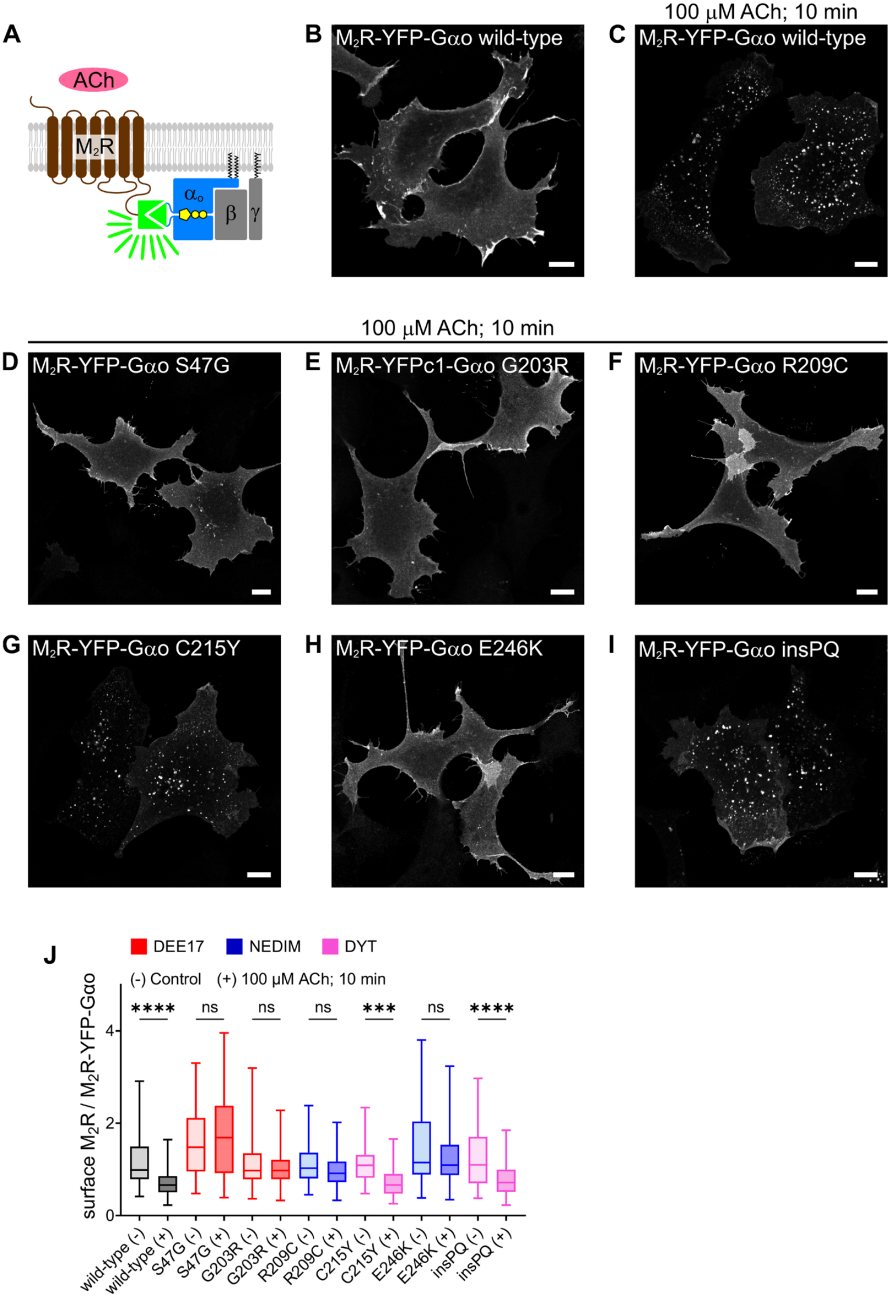
Clinically severe G*α*o mutants disrupt M_2_R endocytosis. (A) Illustration of the split‐YFP assay applied to M_2_R and heterotrimeric G*α*o*β*γ. (B,C) Representative confocal images of HEK293T cells expressing the M_2_R‐YFP‐G*α*o complex at steady state (B) and after 10 min of acetylcholine (ACh) stimulation (C). (D‐I) Confocal images of ACh‐stimulated HEK293T cells expressing the M_2_R‐YFP‐G*α*o complex containing the G*α*o mutants S47G (D), G203R (E), R209C (F), C215Y (G), E246K (H), and insPQ (I). Scale bars, 10 μm. (J) Quantification of ACh‐mediated M_2_R internalization, expressed as the surface level of M_2_R normalized to total M_2_R‐YFP‐G*α*o signal. G*α*o mutant associations with DEE17, NEDIM and DYT phenotypes are color‐coded. Box plots indicate the median (middle line), the 25th and 75th percentiles (box), and the lowest and highest values (whiskers); two‐three independent experiments (wild‐type, *n* = 78; wild‐type +ACh, *n* = 85; S47G, *n* = 55; S47G + ACh, *n* = 51; G203R, *n* = 59; G203R + ACh, *n* = 69; R209C, *n* = 78; R209C + ACh, *n* = 76; C215Y, *n* = 59; C215Y + ACh, *n* = 74; E246K, *n* = 63; E264K + ACh, *n* = 65; insPQ, *n* = 54; insPQ + ACh, *n* = 63). Statistical analysis was performed using one‐way ANOVA followed by Šídák's multiple comparisons test; ***p < 0.001, ****p < 0.0001, and ns: Not significant.

We first generated M_2_R and G*α*o constructs carrying each of the YFP fragments—N‐terminal (YN) or C‐terminal (YC)—fusing them to the intracellular C‐terminus of the receptor and internally at Gly92 in G*α*o (Figure 1A). One of the two possible combinations—M_2_R‐YN and G*α*o‐YC (M_2_R‐YFP‐G*α*o; Figure 1B) – produced a much stronger fluorescence signal than the opposite configuration (not shown), confirming that the orientation of the YFP halves is critical for efficient complementation [41]. As speculated, the M_2_R‐YFP‐G*α*o complex was strongly visualized at the PM of HEK293T cells, with only a few intracellular structures visible (Figure 1B). Notably, acetylcholine (ACh) stimulation for 10 min led to the prominent appearance of vesicles/endosomes and a marked reduction in PM signal, suggesting endocytosis of the M_2_R‐YFP‐G*α*o complex (Figure 1C). Although G*α*o typically remains at PM following GPCR stimulation [30, 42], the irreversibility of split‐YFP complementation likely drives its co‐internalization with the receptor [29].

Next, we performed the split‐YFP assay for M_2_R and several pathogenic G*α*o mutants. We selected three of the most recurrent G*α*o variants—G203R, R209C, and E246K—that may act as DN, either through nonproductive GPCR coupling [21, 23] or via G*β*γ sequestration [22]. We also included the S47G mutant, which shows normal to high GPCR coupling but poor G*β*γ dissociation [21, 23], and has alternatively been described as non‐functional [22]. Among these, G*α*o S47G and G203R are associated with the most severe DEE17 phenotype, while R209C and E246K are linked to NEDIM. Two G*α*o mutants associated with a milder dystonia (DYT) phenotype were also analyzed: C215Y and the recurrent splice‐site variant c.724‐8G > A, which results in an in‐frame insertion of two additional residues (Pro‐Gln) at position T241 (T241_N242insPQ; hereafter insPQ) [18, 23, 27].

As shown in Figure 1, split‐YFP complementation was achieved for all pathogenic G*α*o mutants. However, ACh stimulation revealed striking differences, with DEE17 and NEDIM variants strongly impairing internalization of the M_2_R‐YFP‐G*α*o complex and DYT mutants showing no apparent defects (Figure 1D–I). With longer ACh stimulation, the wild‐type M_2_R‐YFP‐G*α*o complex accumulated on larger endosomes at 20 and 30 min (Figure S2A,B), structures resembling early/late endosomes [43]. In contrast, complexes formed by the DEE17 and NEDIM variants largely remained at the PM even after 30 min, indicating that ACh‐induced M_2_R internalization is blocked rather than merely delayed by the severe G*α*o mutants (Figure S2D–I).

To quantify endocytosis, we analyzed the PM localization of M_2_R‐YN before and after ACh stimulation using immunostaining—under non‐permeabilizing conditions—against an extracellular HA‐epitope at the N‐terminus of the construct (Figure S3A–G). The extracellular HA‐signal was normalized to the intrinsic fluorescence of the M_2_R‐YFP‐G*α*o complex to account for variability in expression levels across cell populations. As expected, a significant reduction of the relative PM pool of M_2_R was observed only for wild‐type G*α*o and the mild DYT variants C215Y and insPQ (Figure 1J). For these G*α*o mutants, we noticed that the HA‐derived M_2_R signal was strongly reduced upon ACh stimulation in some cells, but not in others—particularly those with higher expression levels (Figure S3A–G). This variability may reflect saturation of the receptor endocytic pathway due to overexpression [44].

We also noticed that the overall YFP signal in cells expressing M_2_R‐YFP‐G*α*o complexes was reduced for some of the pathogenic variants compared to wild‐type. Quantification revealed that the DEE17 S47G, NEDIM E246K, and DYT insPQ variants formed significantly lower levels of M_2_R‐YFP‐G*α*o complexes (Figure S3H). This is likely the outcome of reduced expression of the corresponding G*α*o‐YC constructs (Figure S3I,J), consistent with the known tendency of many pathogenic G*α*o mutants to express at lower levels [22, 23]. These expression differences, however, do not account for the defective internalization observed for DEE17 and NEDIM variants, as the DYT insPQ variant—despite a similar reduction in M_2_R‐YFP‐G*α*o formation—underwent robust internalization.

The most straightforward explanation for the failure of M_2_R‐YFP‐G*α*o complexes formed by DEE17/NEDIM variants to undergo endocytosis is a defect in receptor‐G*α*o uncoupling, which prevents progression toward receptor internalization. However, we cannot exclude the possibility that these severe G*α*o mutants may impair endocytosis more broadly. To address this, we examined the internalization of an unrelated receptor, epidermal growth factor receptor (EGFR), using fluorescently labeled EGF‐Rhodamine as readout [35]. EGF‐Rhodamine uptake in HEK293T cells expressing the M_2_R‐YFP‐G*α*o complex was comparable to that in non‐transfected neighboring cells (Figure S4A–G). Furthermore, quantification of internalized EGF‐Rhodamine in cells expressing M_2_R‐YFP‐G*α*o revealed no significant differences among the various G*α*o variants (Figure S4H). These results suggest that expression of DEE17/NEDIM G*α*o mutants does not broadly interfere with receptor endocytosis.

Next, we tested the specificity of the split‐YFP assay using different GPCRs. We selected the μ‐opioid receptor (MOR; Figure S5A), which is known to engage G*α*o, and the *β*_2_‐adrenoceptor (*β*_2_AR; Figure S6A), which signals primarily through G*α*s [45, 46]. Notably, split‐YFP complementation was effectively achieved between wild‐type G*α*o‐YC and either MOR‐YN or *β*_2_AR‐YN, with MOR‐YFP‐G*α*o (Figure S5B) and *β*_2_AR‐YFP‐G*α*o (Figure S6B) complexes localized primarily at the PM of HEK293T cells. These results indicate that split‐YFP complementation with G*α*o is not selective for Gi/o‐coupled receptors. Recapitulating the pattern observed with M_2_R, stimulation with fentanyl for 10 min induced internalization of the wild‐type MOR‐YFP‐G*α*o complex into vesicles/endosomes (Figure S5C), a pattern also observed for the DYT variants, but not for the mutants associated with DEE17/NEDIM (Figure S5D–I). Conversely, isoproterenol treatment induced the endocytosis of the *β*_2_AR‐YFP‐G*α*o complex formed by the wild‐type protein and all pathogenic variants alike (Figure S6C–I), suggesting that the dominant GPCR coupling of G*α*o mutants is specific for Gi/o‐coupled receptors. This finding further implies that split‐YFP complementation between *β*_2_AR and G*α*o does not block the ability of the receptor to couple with endogenous G proteins.

### 3.2 ACh‐Mediated M_2_R Phosphorylation Is Blocked by Dominant G*α*o Variants in the Split‐YFP Assay

Following the dissociation of G proteins from activated GPCRs, a cascade of molecular events is initiated. GPCR kinases (GRKs) are recruited to the receptors, where they bind to the same intracellular pocket previously occupied by the G*α* subunit [47]. GRK recruitment enables phosphorylation of multiple residues within the intracellular loops and/or C‐terminus of the GPCR. This promotes the subsequent recruitment of *β*‐arrestins, which act as scaffolds for the clathrin‐mediated endocytic machinery, ultimately driving receptor internalization [48].

Our findings support the notion that the most severe pathogenic G*α*o variants fail to disengage from activated GPCRs, remaining dominantly coupled and thereby preventing GRK recruitment and subsequent receptor phosphorylation. To test this prediction, we analyzed M_2_R phosphorylation using the split‐YFP assay. We used a non‐pathogenic G*α*o construct carrying a four‐alanine insertion in the C‐terminal *α*5‐helix (ins4A) as control, which is known to abolish G protein release from activated receptors [49, 50]. As expected, G*α*o ins4A prevented ligand‐mediated internalization of both M_2_R Figure S7A–C and MOR Figure S7D–F in the split‐YFP assay.

**FIGURE 2:**
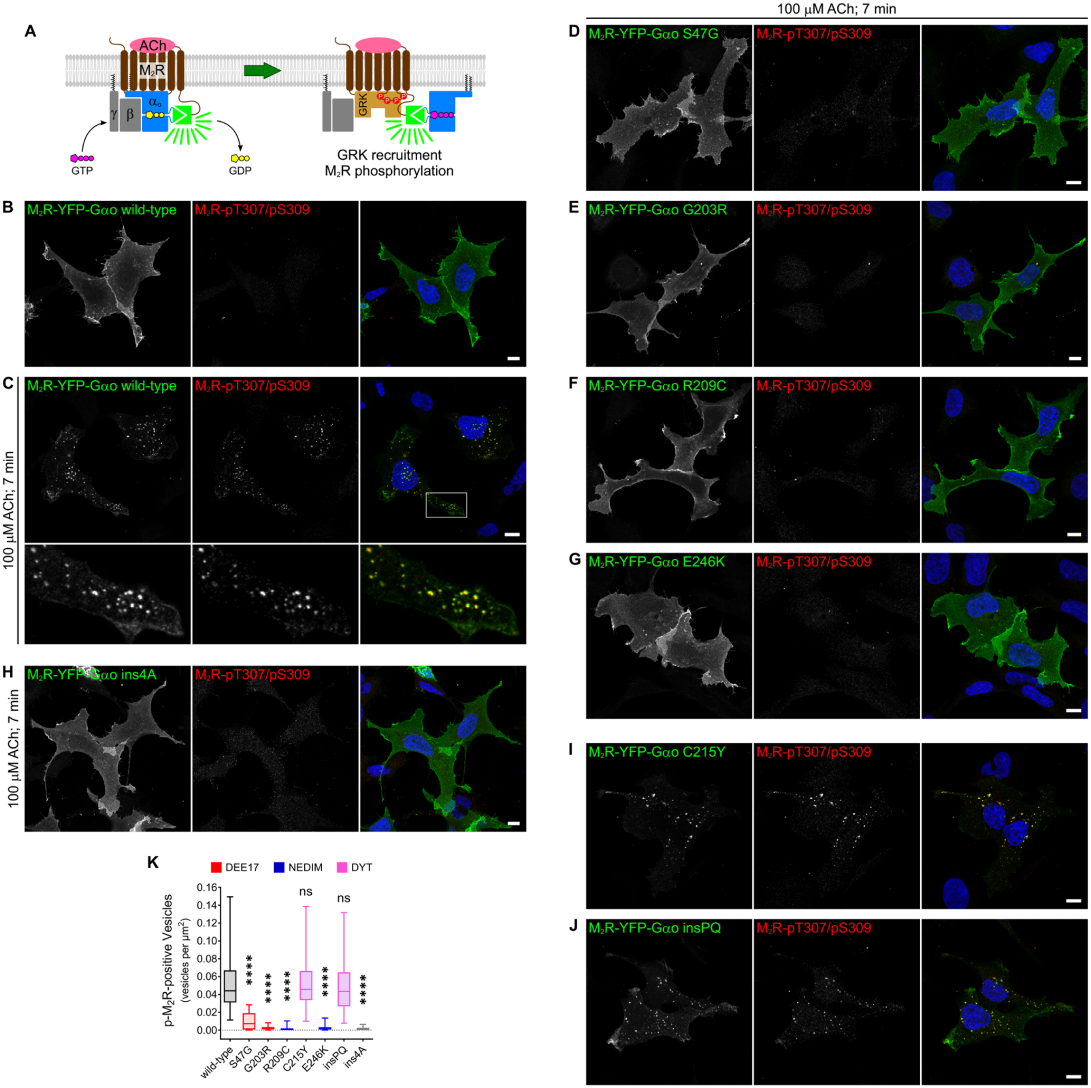
Inhibition of ACh‐mediated phosphorylation of M_2_R by pathogenic G*α*o variants. (A) Schematic representation of the molecular events following ACh stimulation that lead to M_2_R phosphorylation, as illustrated using the split‐YFP assay. (B,C) Representative confocal images of HEK293T cells expressing the M_2_R‐YFP‐G*α*o complex at steady state (B) and after 7 min of acetylcholine (ACh) stimulation (C). Cells were immunostained with a specific antibody recognizing dual phosphorylation at T307 and S309 of M_2_R (M_2_R‐pT307/pS309), and counterstained with DAPI to visualize nuclei. A boxed region in (C) is shown at higher magnification in the lower panels. (D‐H) ACh‐stimulated HEK293T cells expressing the M_2_R‐YFP‐G*α*o complex with the G*α*o mutants S47G (D), G203R (E), R209C (F), E246K (G), C215Y (I), insPQ (J), and the control ins4A (H). Cells were prepared as in (C). Scale bars, 10 μm. (K) Quantification of ACh‐mediated endocytic events, expressed as the number of vesicle‐like structures positive for both M_2_R‐YFP‐G*α*o and anti‐pT307/pS309 staining, normalized to cell area. Associations of G*α*o mutants with DEE17, NEDIM, and DYT phenotypes are color‐coded. Box plots indicate the median (middle line), the 25th and 75th percentiles (box), and the lowest and highest values (whiskers); two‐three independent experiments (wild‐type, *n* = 46; S47G, *n* = 43; G203R, *n* = 46; R209C, *n* = 44; C215Y, *n* = 48; E246K, *n* = 47; insPQ, *n* = 50; ins4A, *n* = 46). Statistical analysis was performed using one‐way ANOVA followed by Dunnett's multiple comparisons test; ****p < 0.0001 and ns: Not significant.

GRK‐mediated phosphorylation of M_2_R targets several Ser/Thr residues in the third intracellular loop (Figure 2A) [51]. We immunostained HEK293T cells expressing the M_2_R‐YFP‐G*α*o complex using a specific antibody that recognizes dual phosphorylation at Thr307 and Ser309 (pT307/pS309). In unstimulated cells, the anti‐pT307/pS309 signal was minimal in both transfected and non‐transfected cells (Figure 2B). However, following 7 min of ACh stimulation, robust anti‐pT307/pS309 staining appeared and closely colocalized with endocytic M_2_R‐YFP‐G*α*o–positive vesicles (Figure 2C). Neighboring untransfected cells showed no increase in the pT307/pS309 signal after stimulation, consistent with the absence of endogenous M_2_R expression in the parental HEK293 line [52].

Notably, the G*α*o variants associated with the severe DEE17/NEDIM phenotypes exhibited weak pT307/pS309 staining following ACh stimulation (Figure 2D–G). Since the ins4A construct produced an equivalent effect (Figure 2H), this result supports the notion that G*α*o mutants dominantly couple to M_2_R, thereby preventing GRK recruitment. In contrast, the DYT‐associated mutants retained robust phospho‐M_2_R staining, similar to wild‐type G*α*o (Figure 2I,J). To quantify endocytic events, we counted the number of vesicle‐like structures positive for both M_2_R‐YFP‐G*α*o and pT307/pS309 and normalized to the cell area (Figure 2K). A marked reduction in phospho‐M_2_R‐YFP‐G*α*o–positive vesicles was observed for the DEE17/NEDIM variants and ins4A, whereas DYT mutants exhibited values comparable to wild‐type.

To confirm the defects in ACh‐mediated M_2_R phosphorylation, we took advantage of an anti‐GFP nanobody [31] that enables specific immunoprecipitation (IP) of the reconstituted split‐YFP, but not the individual YN and YC fragments (Figure S8). In Western blots following IPs, four distinct protein species can be detected using M2R‐ and G*α*o‐specific antibodies: the M_2_R‐YN and G*α*o‐YC monomers, a monomeric M_2_R‐YFP‐G*α*o complex, and a higher molecular weight species, possibly corresponding to a M_2_R‐YFP‐G*α*o dimer (Figure S8). This likely reflects the known tendency of M_2_R to form dimers and higher‐order oligomers [53].

**FIGURE 3:**
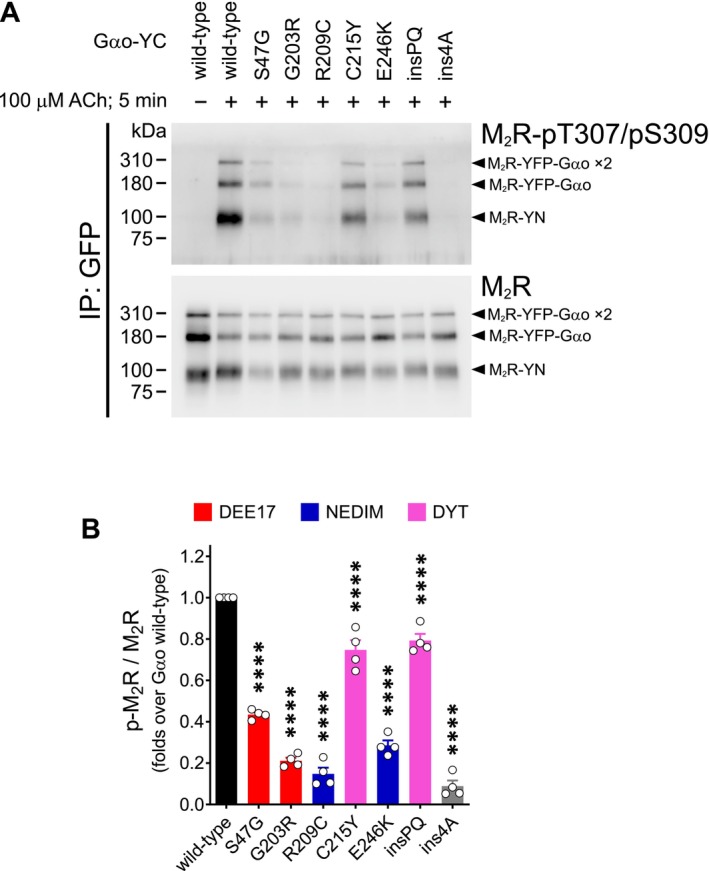
Immunoprecipitation of the M_2_R‐YFP‐G*α*o complex confirms impaired recephosphorylation by pathogenic G*α*o variants. (A) HEK293T cells expressing the split‐YFP constructs M_2_R‐YN and G*α*o‐YC (wild‐type, pathogenic mutants, and the ins4A control) were stimulated with 100 μM acetylcholine (ACh) for 5 min and subjected to immunoprecipitation (IP) using an anti‐GFP nanobody. Western blotting and immunodetection were performed with antibodies against phosphorylated (pT307/pS309) and total M_2_R. Arrowheads indicate M_2_R‐YN, and the monomeric and dimeric forms of the M_2_R‐YFP‐G*α*o complex. (B) Quantification of M_2_R phosphorylation levels relative to total receptor (*n* = 4). G*α*o mutant associations with DEE17, NEDIM, and DYT phenotypes are color‐coded. Data represent mean ± SEM. Statistical analysis was performed using one‐way ANOVA followed by Dunnett's multiple comparisons test; ****p < 0.0001.

Corroborating the cell imaging data, IP of split‐YFP complexes containing wild‐type G*α*o showed no detectable anti‐pT307/pS309 signal for M_2_R at steady state, whereas strong signals emerged following 5 min of ACh stimulation in three species: M_2_R‐YN, the monomeric, and the dimeric forms of the M_2_R‐YFP‐G*α*o complex (Figure 3A). In contrast, M_2_R‐YFP‐G*α*o complexes formed by the most severe DEE17/NEDIM mutants exhibited a marked reduction in anti‐pT307/pS309 reactivity. Unexpectedly, both DYT‐associated G*α*o variants showed a modest reduction in M_2_R phosphorylation. Quantification revealed a 60%–85% reduction for the DEE17/NEDIM variants, and over 90% for G*α*o ins4A (Figure 3B). DYT mutants, on the other hand, showed a significant but smaller 20%–25% decrease, suggesting that these variants partially impair GRK recruitment—although not to a degree that prevents receptor endocytosis.

### 3.3 Untagged G*α*o Mutants Recapitulate M_2_R Endocytosis and Phosphorylation Defects

While the split‐YFP assay provides a powerful tool to visualize these interactions, it relies on internally tagged G*α*o constructs, which could theoretically introduce artifacts not present in native, untagged proteins. To address this, we next tested whether the observed effects also occur with untagged G*α*o variants.

**FIGURE 4:**
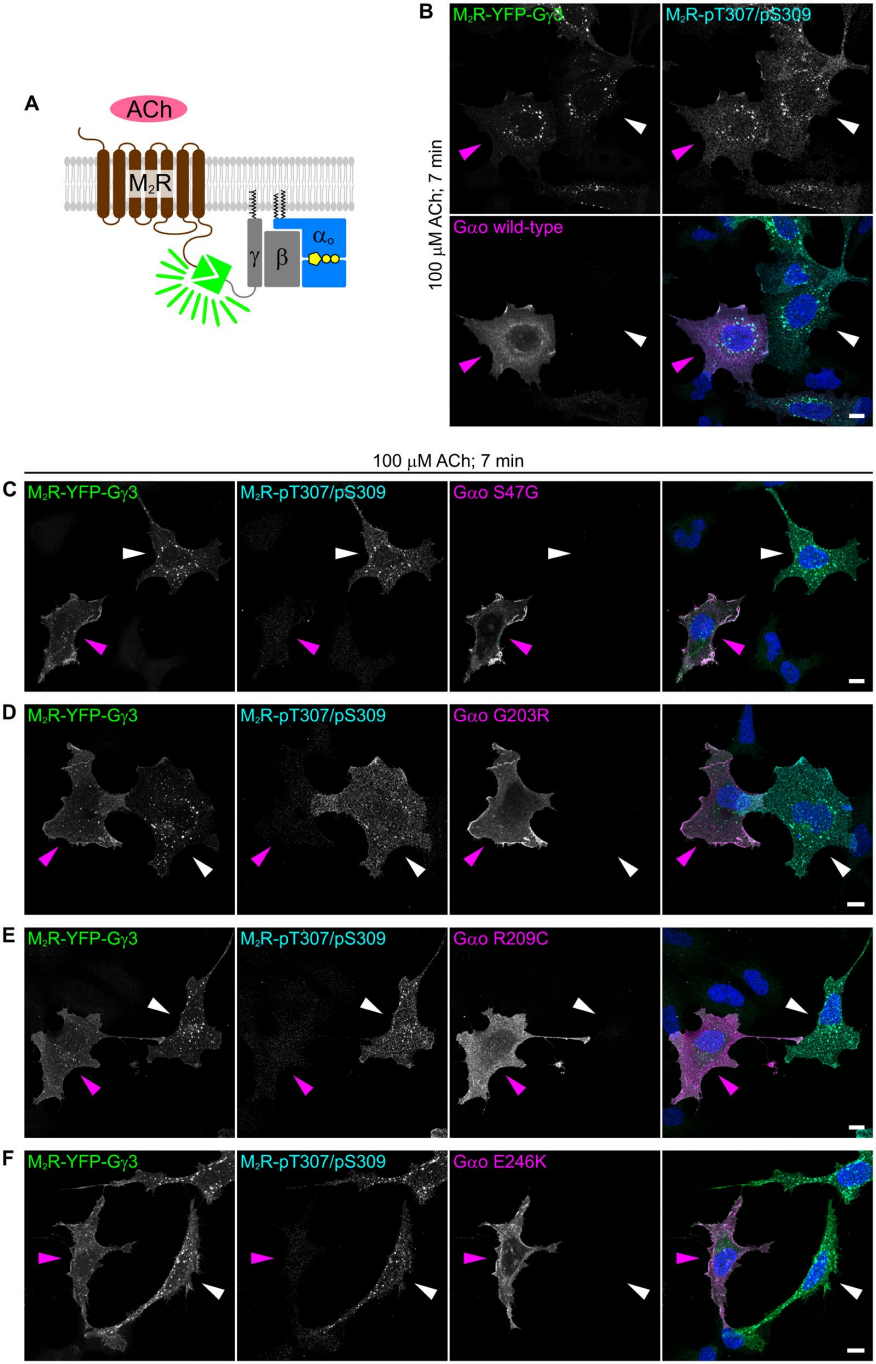
Split‐YFP assay with M_2_R and Gγ3 recapitulates phosphorylation and endocytosis defects caused by G*α*o mutants. (A) Depiction of the split‐YFP assay applied to M_2_R and Gγ3. (B‐F) Confocal images of co‐culture of HEK293T cells expressing the M_2_R‐YFP‐Gγ3 complex, with or without co‐expression of G*α*o wild‐type (B), S47G (C), G203R (D), R209C (E), and E246K (F). Cells were stimulated with 100 μM acetylcholine (ACh) for 7 min, then immunostained with anti‐pT307/pS309, anti‐G*α*o, and counterstained with DAPI to visualize nuclei. Magenta and white arrowheads indicate G*α*o‐expressing and non‐expressing cells, respectively. Scale bars, 10 μm.

We first employed the split‐YFP assay for M_2_R and Gγ3 (Figure 4A), and found that the combination of M_2_R‐YC and YN‐Gγ3 produced a much stronger fluorescence signal than the reverse configuration (not shown). To test dominant GPCR coupling and receptor endocytosis, we expressed different construct sets in two separate HEK293T cell populations—M_2_R‐YC and YN‐Gγ3 with or without untagged G*α*o—and then co‐cultured them. After 7 min of ACh treatment, cells were immunostained for G*α*o and pT307/pS309. Anti‐G*α*o signal was absent in non‐transfected HEK293T cells, consistent with the lack of G*α*o expression in the parental HEK293 line [52]. ACh stimulation triggered robust internalization of the M_2_R‐YFP‐Gγ3 complex in the presence or absence of wild‐type G*α*o expression, suggesting that endogenous G*α* subunits were sufficient to engage the receptor (Figure 4B). Notably, co‐expression of the DEE17/NEDIM variants markedly inhibited both M_2_R phosphorylation and endocytosis, contrasting with the normal response observed in adjacent G*α*o‐negative cells (Figure 4C–F).

**FIGURE 5:**
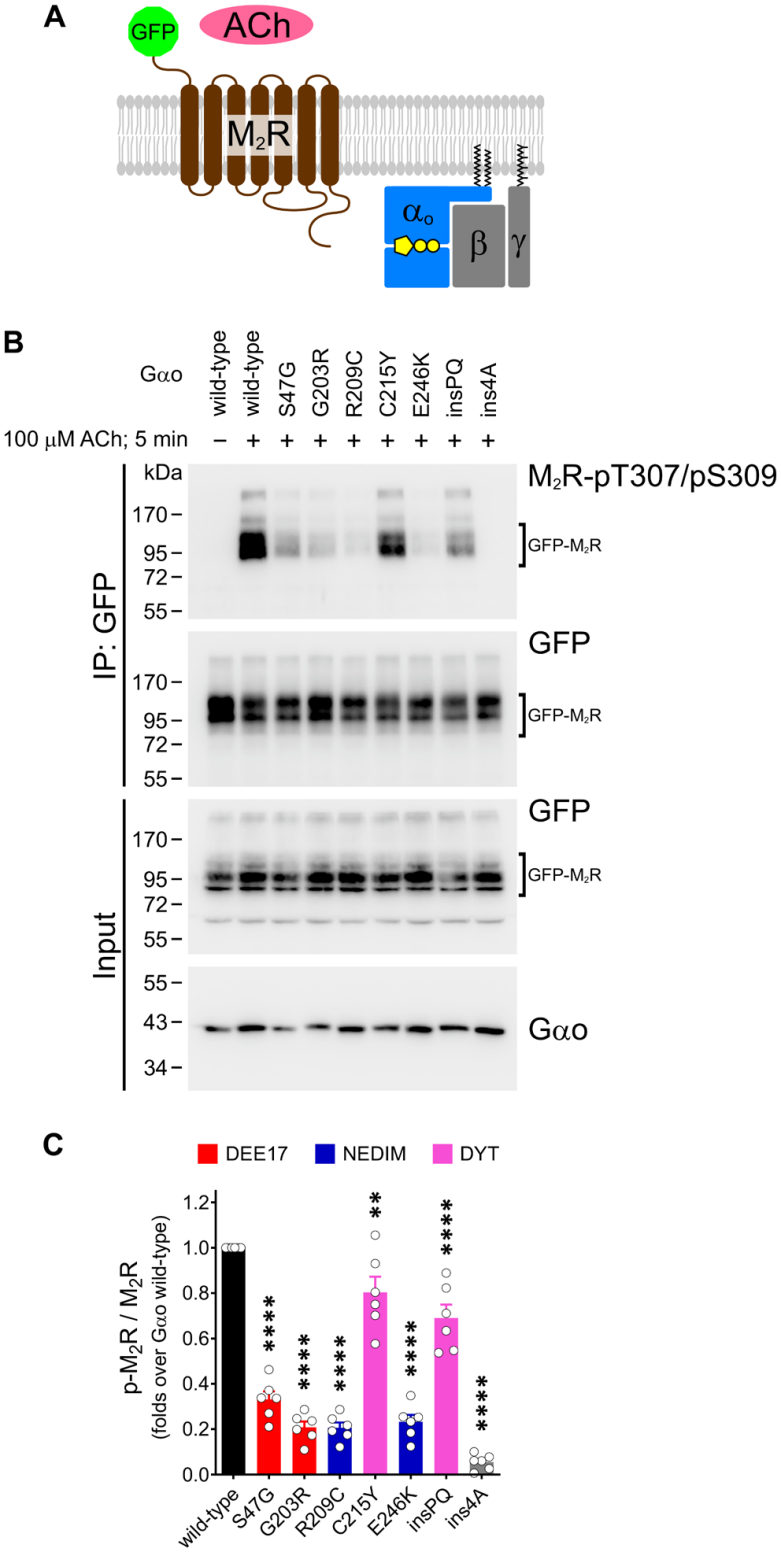
Immunoprecipitation of GFP‐M_2_R validates dominant GPCR coupling by pathogenic G*α*o variants. (A) Diagram of the GFP‐M_2_R construct and heterotrimeric G*α*o*β*γ, illustrating the lack of intracellular tagging in the proteins. (B) HEK293T cells co‐expressing GFP‐M_2_R and G*α*o wild‐type, pathogenic mutants, or the ins4A control were stimulated with 100 μM acetylcholine (ACh) for 5 min and subjected to immunoprecipitation (IP) using an anti‐GFP nanobody. Western blotting and immunodetection were performed with antibodies against pT307/pS309 and GFP. (C) Quantification of M_2_R phosphorylation levels relative to total receptor (*n* = 6). G*α*o mutant associations with DEE17, NEDIM, and DYT phenotypes are color‐coded. Data represent mean ± SEM. Statistical analysis was performed using one‐way ANOVA followed by Dunnett's multiple comparisons test; **p < 0.005 and ****p < 0.0001.

Finally, we moved beyond the split‐YFP assay and analyzed ACh‐mediated phosphorylation of GFP‐M_2_R upon co‐expression of untagged G*α*o variants (Figure 5A). We selected the GFP‐M_2_R construct because it lacks intracellular tags, thereby avoiding potential artifacts originating from the receptor. We first examined whether the PM localization of GFP‐M_2_R was affected by any G*α*o variant. To this end, we immunostained the extracellular M_2_R pool under non‐permeabilizing conditions using an anti‐GFP antibody and normalized the signal to the total GFP fluorescence to account for differences in expression (Figure S9A–H). Quantification showed that GFP‐M_2_R surface targeting was unaffected by co‐expression of G*α*o variants (Figure S9I).

As expected, IP analysis revealed no detectable phosphorylation of GFP‐M_2_R at steady state, whereas a strong anti‐pT307/pS309 signal was observed following 5 min of ACh stimulation (Figure 5B). Remarkably, all pathogenic G*α*o mutants reduced GFP‐M_2_R phosphorylation, following a pattern closely resembling that observed with the split‐YFP assay. Quantification revealed a pronounced 65%–80% reduction in M_2_R phosphorylation upon co‐expression of the DEE17/NEDIM variants, and ~95% reduction for the G*α*o ins4A mutant (Figure 5C). In contrast, co‐expression of the DYT variants C215Y and insPQ led to more modest reductions of 20% and 30%, respectively.

Altogether, this study indicates that pathogenic G*α*o variants remain persistently and dominantly coupled to activated GPCRs. These findings highlight a critical pathogenic mechanism in *GNAO1* encephalopathies and establish a robust split‐YFP assay for its detection and potential therapeutic targeting.

## 4 Discussion

Pathogenic *GNAO1* mutations disrupt multiple aspects of G*α*o function [21, 22, 23]. Two distinct mechanisms—nonproductive GPCR coupling and G*β*γ sequestration—have been independently reported for several G*α*o variants [21, 22, 23]. This apparent contradiction, together with the absence of a unified biosensor approach, prompted us to develop a split‐YFP assay to directly visualize the dominant GPCR coupling behavior of G*α*o mutants. Using this system, we found that persistent, dominant coupling to activated GPCRs is a common feature among pathogenic G*α*o variants. The most severe mutants failed to disengage from activated receptors, thereby blocking GRK‐mediated phosphorylation and subsequent endocytosis. In contrast, less severe mutants only partially impaired GPCR phosphorylation without preventing internalization.

BiFC assays have been widely used to analyze protein–protein interactions in living cells [29], including studies on GPCRs, where most applications have focused on receptor homo‐ and heteromerization [38, 54, 55]. Other BiFC‐based approaches have visualized G*β*γ dimer formation and localization [56], Src association with GPCRs [38], and *β*‐arrestin recruitment following receptor activation [57, 58]. To our knowledge, this study represents the first BiFC system specifically designed to monitor GPCR coupling by a G*α* subunit in mammalian cells. In this assay, YFP complementation at the PM between GPCRs and the G*α* appears to be nonselective, as G*α*o was able to efficiently tether to the Gs‐coupled *β*_2_AR. Upon stimulation, the assay appears highly specific, since pathogenic G*α*o variants disrupted internalization of Gi/o‐coupled GPCRs but had no effect on *β*_2_AR endocytosis. The irreversibility of the YFP complementation [29] limits the ability of this system to analyze events downstream of G protein activation, as the complemented G*α* subunit remains stably associated with the GPCR. This same property, however, allowed us to readily isolate the complemented split‐YFP complex using an anti‐GFP nanobody [31], enabling biochemical characterization of the GPCR‐YFP‐G*α* complex and potentially opening opportunities for structural studies of dominant GPCR coupling mediated by pathogenic G*α*o variants.

In this study, we used HEK293T cells because they allow robust expression of the split‐YFP constructs, making the BiFC assay feasible. The non‐neuronal nature of this cell line, however, raises the question of whether pathogenic G*α*o variants also engage in dominant GPCR coupling under more physiological conditions. Therefore, further studies will be required to confirm this dominant mechanism in neuronal systems, for example using cortical neurons derived from *GNAO1* patient iPSCs as described previously [59].

**FIGURE 6:**
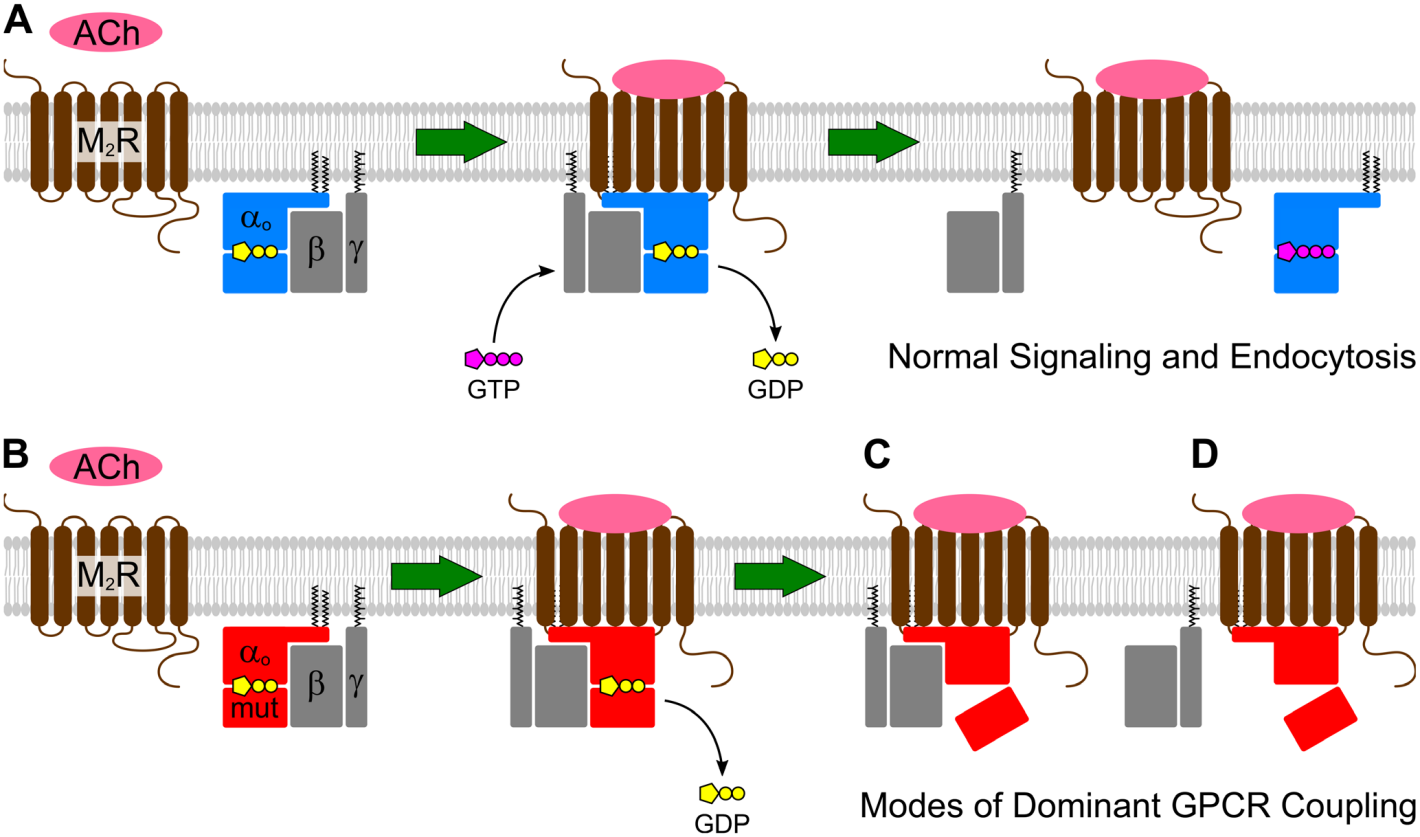
Proposed mechanisms of dominant GPCR coupling by pathogenic G*α*o mutants. (A) Under physiological conditions, acetylcholine (ACh) activates the M_2_ muscarinic acetylcholine receptor (M₂R), promoting GDP‐GTP exchange on wild‐type G*α*o (blue) within the heterotrimeric G protein. This activation triggers dissociation of G*α*o‐GTP and G*β*γ subunits, initiating downstream signaling and allowing receptor phosphorylation and endocytosis. (B‐D) Pathogenic G*α*o variants (red) engage activated GPCRs leading to GDP release (B). The failure of G*α*o mutants to adopt the active GTP‐bound conformation results in dominant GPCR coupling through two distinct mechanisms. In one mode, the mutant G*α*o remains in an open, intermediate activation state that retains both the GPCR and G*β*γ within a persistent heterotrimeric complex (C). In the second mode, mutant G*α*o remains tightly bound to the activated receptor in a nucleotide‐free state after G*β*γ dissociation (D). Both mechanisms impair receptor phosphorylation and block endocytosis, potentially leading to signaling defects.

Dominant GPCR coupling by G*α* subunits has long been recognized in the GPCR field [49, 50, 60, 61]. Studies using engineered, non‐pathogenic G*α* mutations have proposed two mechanistic modes: sequestration of the activated GPCR by the heterotrimeric G protein, or by nucleotide‐free G*α* subunits. Although our split‐YFP assay cannot discriminate between these mechanisms, insight can be drawn from the distinct behaviors of G*α*o variants in previous BRET‐based assays. For example, the S47G mutant likely traps the receptor—and G*β*γ—in an open, intermediate activation state of the heterotrimeric complex, as it was reported to retain near‐normal association with G*β*γ but shows markedly impaired dissociation upon GPCR activation [21, 22, 23]. In contrast, the recurrent G203R, R209C, and E246K variants exhibit significant G*β*γ dissociation upon stimulation [21, 22, 23, 62], consistent with receptor trapping by nucleotide‐free G*α*o following G*β*γ dissociation (Figure 6). Nevertheless, confirming that these G*α*o variants indeed trap activated GPCRs and distinguishing between these two modes will require further mechanistic studies.

In addition to G*α*o, dominant GPCR coupling has thus far been implicated only in pathogenic G*α*i3 mutations [63]. However, several residues homologous to the G*α*o positions analyzed here—Ser47, Gly203, Arg209, and Glu246—are also mutated in other G*α* subunits linked to rare genetic conditions [11]: G*α*i2 G203R and R209W, G*α*olf E255A, G*α*s R231C/H and E268K/G, and G*α*i3 S47R, which was shown to dominantly couple to the endothelin type A receptor. These observations support the possibility that dominant GPCR coupling may extend beyond G*α*o and G*α*i3, as many disease‐associated mutations affect additional highly conserved residues shared across G*α* subunits [11, 12]. Thus, we envision that this simple split‐YFP approach could be adapted for routine testing of G*α*o and potentially other pathogenic G*α* variants implicated in a growing number of rare genetic disorders.

Beyond mechanistic insights, the robustness and scalability of the split‐YFP system lay a foundation for drug discovery or repositioning efforts aimed at restoring normal GPCR coupling dynamics. Such applications could accelerate the identification of therapeutic compounds that reverse receptor trapping or promote dissociation of G*α*o mutants, offering new avenues for targeted therapies in *GNAO1* encephalopathies.

## Author Contributions

Conceptualization: G.P.S. Methodology: C.RdM., G.P.S. Investigation: Y.A.L., G.P.S. Visualization: Y.A.L., G.P.S. Writing‐original draft: Y.A.L., G.P.S. Writing‐review and editing: M.S., V.L.K., G.P.S. Supervision: V.L.K., G.P.S. All authors reviewed the final manuscript.

## Funding

The authors have nothing to report.

## Conflicts of Interest

The authors declare no conflicts of interest.

## Supporting information




**Data S1:** Supplementary Figures.

## Data Availability

The data that support the findings of this study are available in the Materials and Methods, Results, and Supporting Information of this article.
