# Supplementary material for: Pathogenic Gαo Mutants Drive Dominant GPCR Coupling in GNAO1 Encephalopathies
Source: FASEB J. 2025 Dec 29;40(1):e71402. doi: 10.1096/fj.202503265R (PMC12746902; doi:10.1096/fj.202503265R)
Supplement: Supplementary file 1 — Data S1: Supplementary Figures. [file FSB2-40-e71402-s001.pdf]

Supplemental Information

Pathogenic Gαo Mutants Drive Dominant GPCR Coupling in *GNAO1* Encephalopathies

Yonika A. Larasati[1,ORCID], Camille Rabesahala de Meritens[1,ORCID], Miriam Stoeber[2,ORCID], Vladimir L. Katanaev[1,3,ORCID] , Gonzalo P. Solis[1,ORCID]

[1] Translational Research Center in Oncohaematology, Department of Cell Physiology and Metabolism, Faculty of Medicine, University of Geneva, Geneva, Switzerland.

[2] Department of Cell Physiology and Metabolism, Faculty of Medicine, University of Geneva, Geneva, Switzerland.

[3] Translational Oncology Research Center, Qatar Biomedical Research Institute (QBRI), College of Health and Life Sciences, Hamad Bin Khalifa University (HBKU), Qatar Foundation, Doha, Qatar.

Correspondence: Vladimir Katanaev (vladimir.katanaev@unige.ch) and Gonzalo Solis (gonzalo.solis@unige.ch). Department of Cell Physiology and Metabolism, CMU, Faculty of Medicine, University of Geneva. Rue Michel-Servet 1. CH-1211 Genève 4, Switzerland. Phone: +41.22.379.53.17

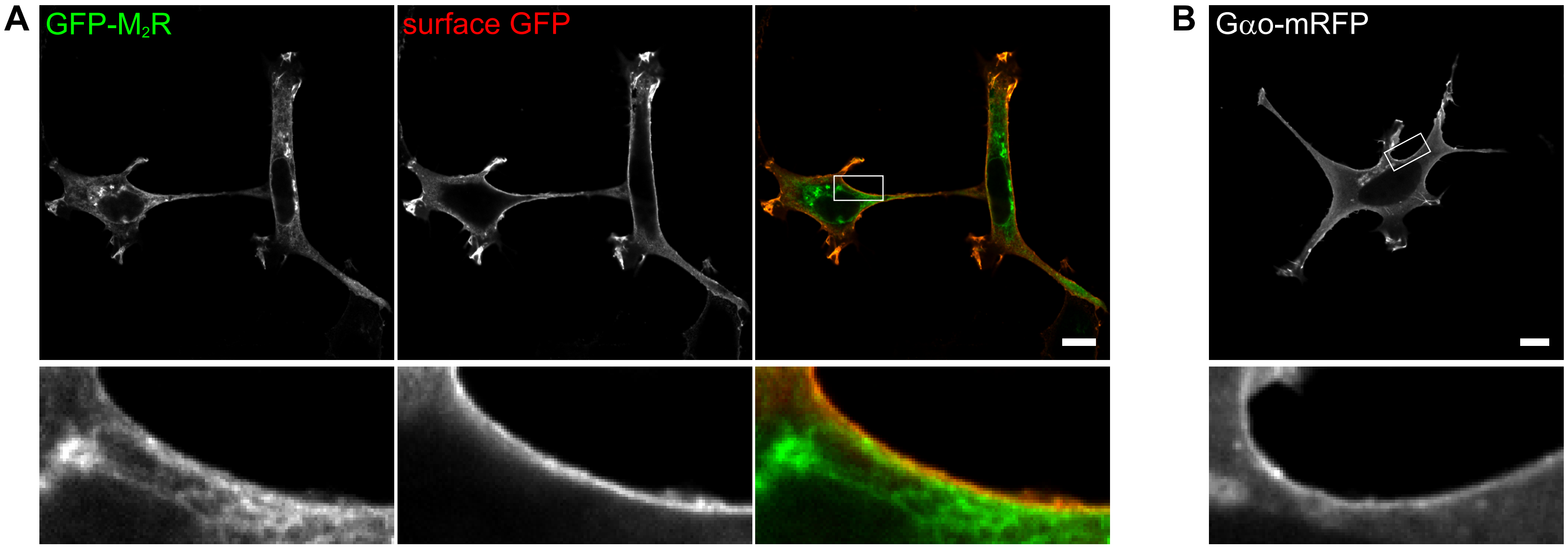

Figure S1. Figure S1. Subcellular localization of M_2R and Gαo in HEK293T cells. (**A**,**B**) Confocal images of HEK293T cells expressing N-terminally GFP-tagged M_2R (GFP-M_2R; **A**) and Gαo internally tagged with mRFP (Gαo-mRFP; **B**). GFP-M_2R-expressing cells were immunostained with anti-GFP under non-permeabilizing conditions to label surface GFP. Boxed regions are shown at higher magnification in the lower panels. Scale bars, 10 µm.

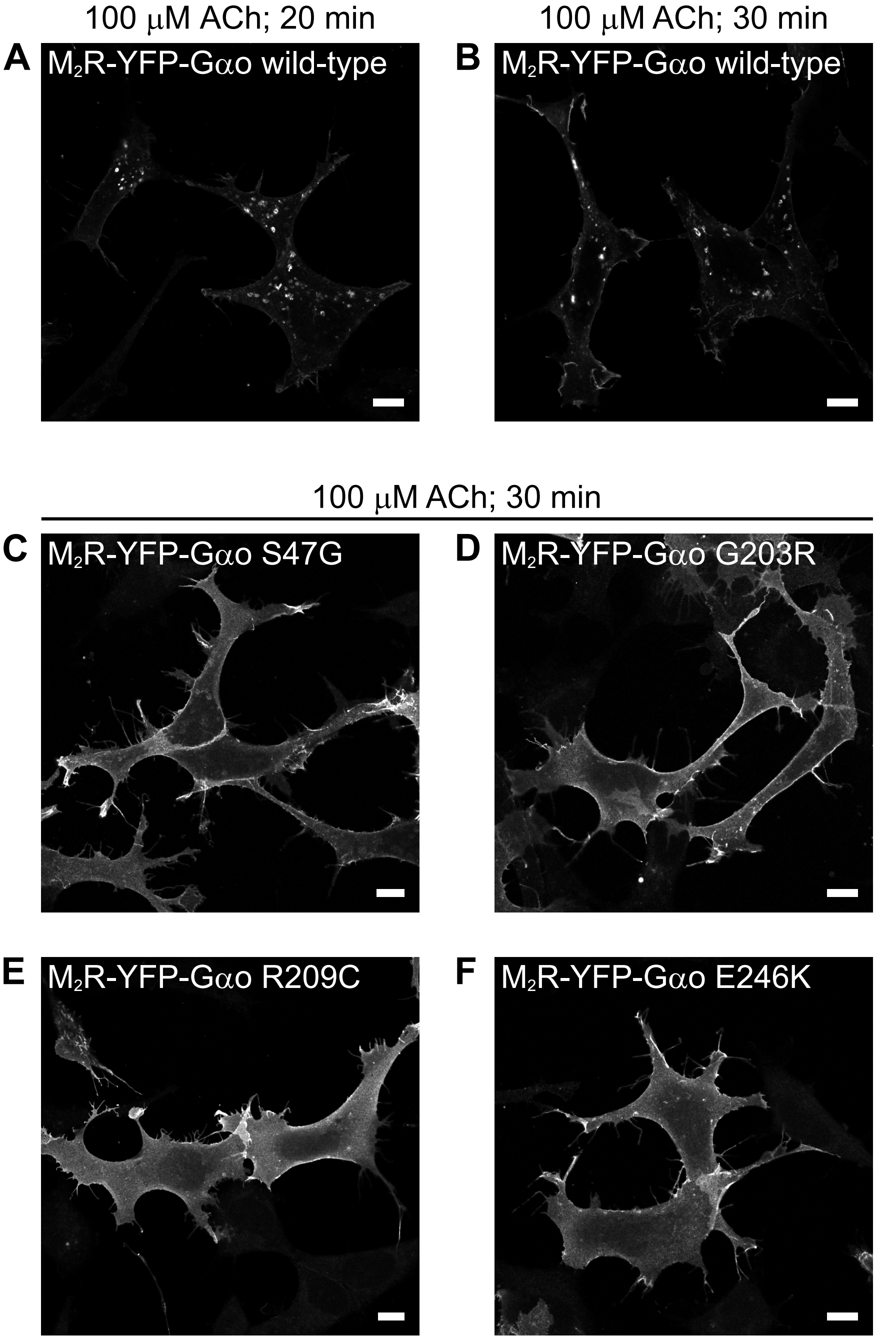

Figure S2. ACh-mediated M_2R endocytosis is blocked by clinically severe Gαo variants. (**A**,**B**) Representative confocal images of HEK293T cells expressing the wild-type M_2R-YFP-Gαo complex after 20 min (**A**) and 30 min of 100 μM ACh stimulation (**B**). (**C**-**F**) Confocal images of ACh-stimulated HEK293T cells expressing the M_2R-YFP-Gαo complex formed with the indicated pathogenic Gαo mutant. Scale bars, 10 μm.

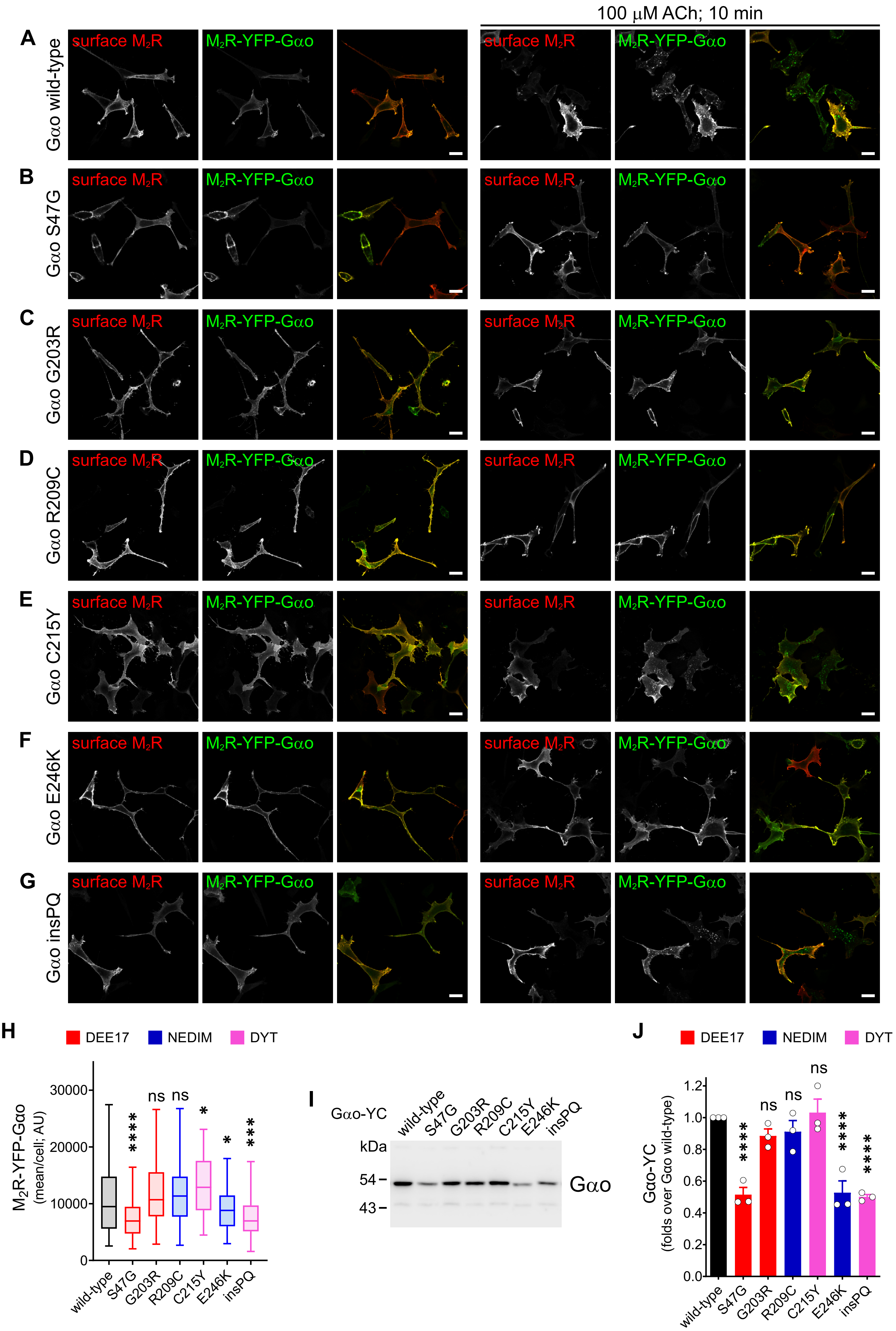

Figure S3. ACh-mediated stimulation of HEK293T cells expressing the M_2R-YFP-Gαo complex. (**A-G**) Representative confocal images of HEK293T cells expressing the M_2R-YFP-Gαo complex containing the indicated Gαo variants. Cells were immunostained under non-permeabilizing conditions to label surface M_2R at steady state (left panels) and after 10 min of acetylcholine (ACh) stimulation (right panels). Scale bars, 10 μm. (**H**) Quantification of M_2R-YFP-Gαo complex formation across different Gαo variants. Gαo mutant associations with DEE17, NEDIM and DYT phenotypes are color-coded. Box plots indicate the median (middle line), the 25th and 75th percentiles (box), and the lowest and highest values (whiskers); two-three independent experiments (wild-type, n = 78; S47G, n = 55; G203R, n = 59; R209C, n = 78; C215Y, n = 59; E246K, n = 63; insPQ, n = 54). (**I**) HEK293T cells expressing the split-YFP Gαo-YC construct (wild-type and pathogenic mutants) were analyzed by Western blot using an anti-Gαo antibody. (**J**) Quantification of Gαo-YC mutant expression levels relative to wild-type (n = 3). Data represent mean ± SEM. Statistical analyses were performed using one-way ANOVA followed by Dunnett's multiple comparisons test; *p < 0.05, ***p < 0.001, ****p < 0.0001, and ns: not significant.

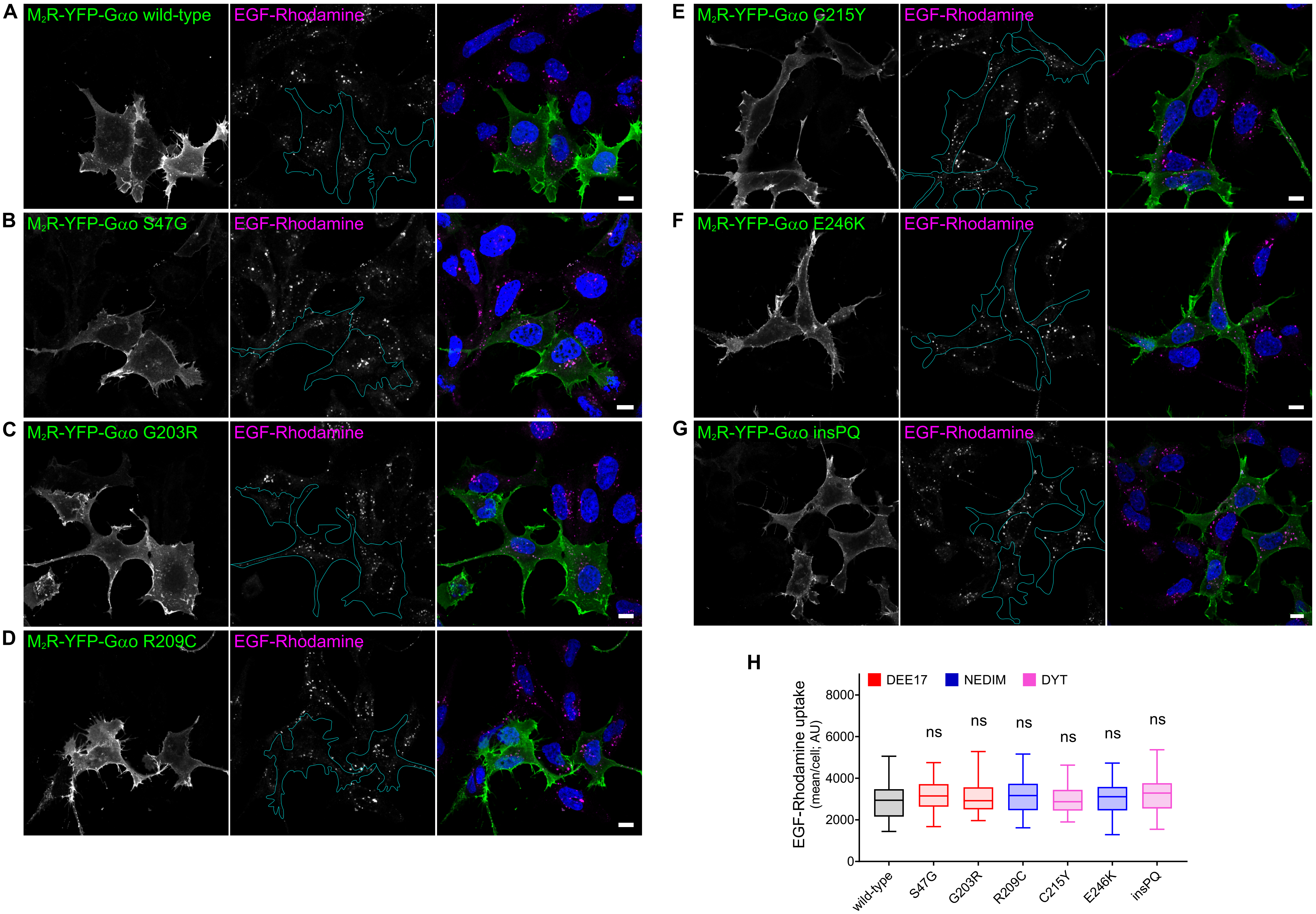

Figure S4. EGF-Rhodamine uptake by HEK293T cells expressing the M$_2$R-YFP-Gαo complex. (**A-G**) Representative confocal images of HEK293T cells expressing the M$_2$R-YFP-Gαo complex with the indicated Gαo variants. Cells were incubated with 10 ng/ml of EGF-Rhodamine for 10 min. Nuclei were visualized by DAPI staining. Cyan lines demarcate cells expressing M$_2$R-YFP-Gαo. Scale bars, 10 µm. (**H**) Quantification of EGF-Rhodamine uptake. Gαo mutant associations with DEE17, NEDIM and DYT phenotypes are color-coded. Box plots indicate the median (middle line), the 25th and 75th percentiles (box), and the lowest and highest values (whiskers); two independent experiments (wild-type, n = 51; S47G, n = 56; G203R, n = 51; R209C, n = 52; C215Y, n = 57; E246K, n = 52; insPQ, n = 55). Statistical analysis was done using one-way ANOVA followed by Dunnett's multiple comparisons test; ns: not significant.

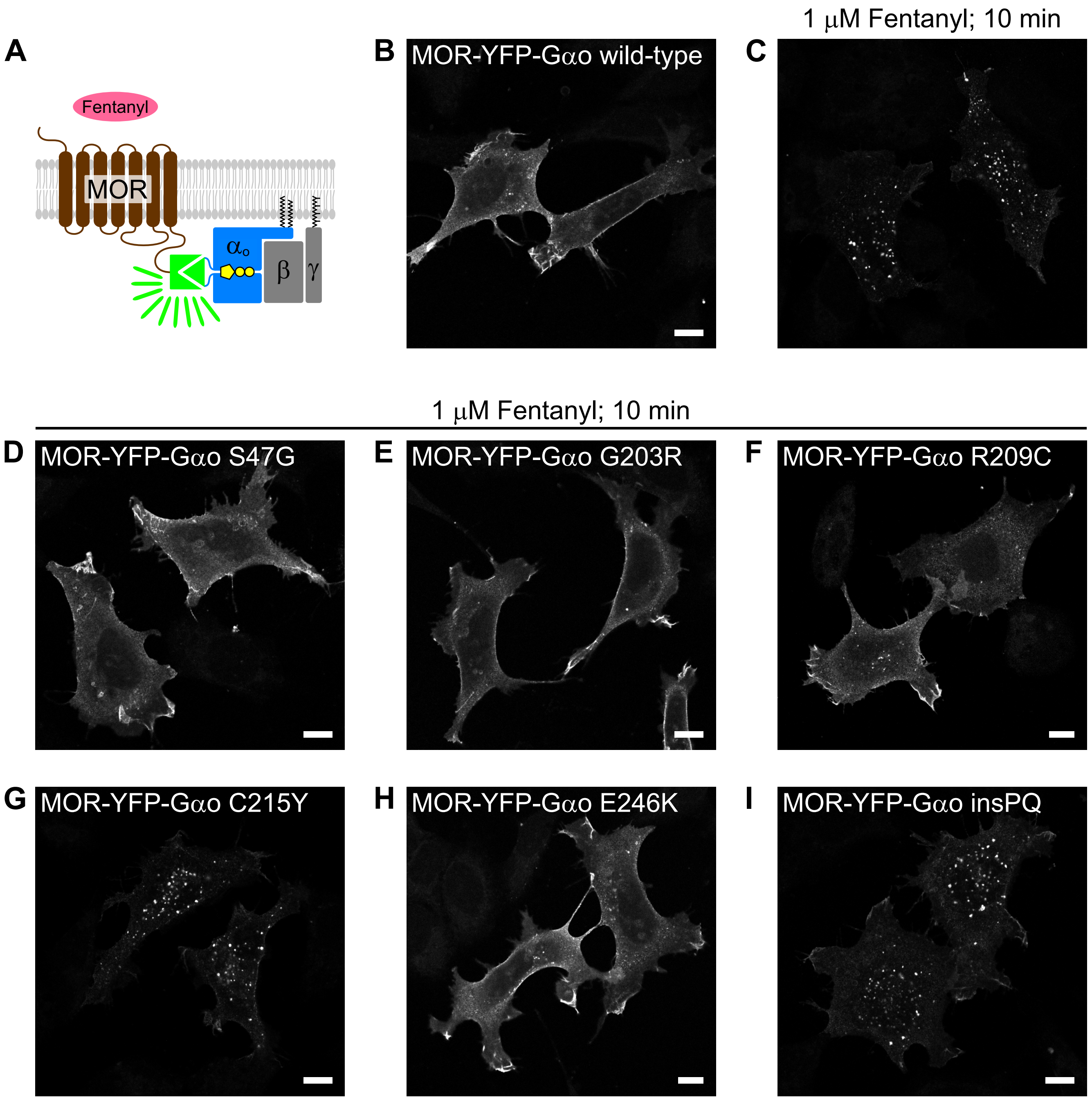

Figure S5. Clinically severe Gαo mutants disrupt μ-opioid receptor (MOR) endocytosis. (**A**) Illustration of the split-YFP assay applied to MOR and heterotrimeric Gαoβγ. (**B**,**C**) Representative confocal images of HEK293T cells expressing the MOR-YFP-Gαo complex at steady state (**B**) and after 10 min of 1 μM fentanyl stimulation (**C**). (**D**-**I**) Confocal images of fentanyl-stimulated HEK293T cells expressing the MOR-YFP-Gαo complex with the indicated pathogenic Gαo variants. Scale bars, 10 μm.

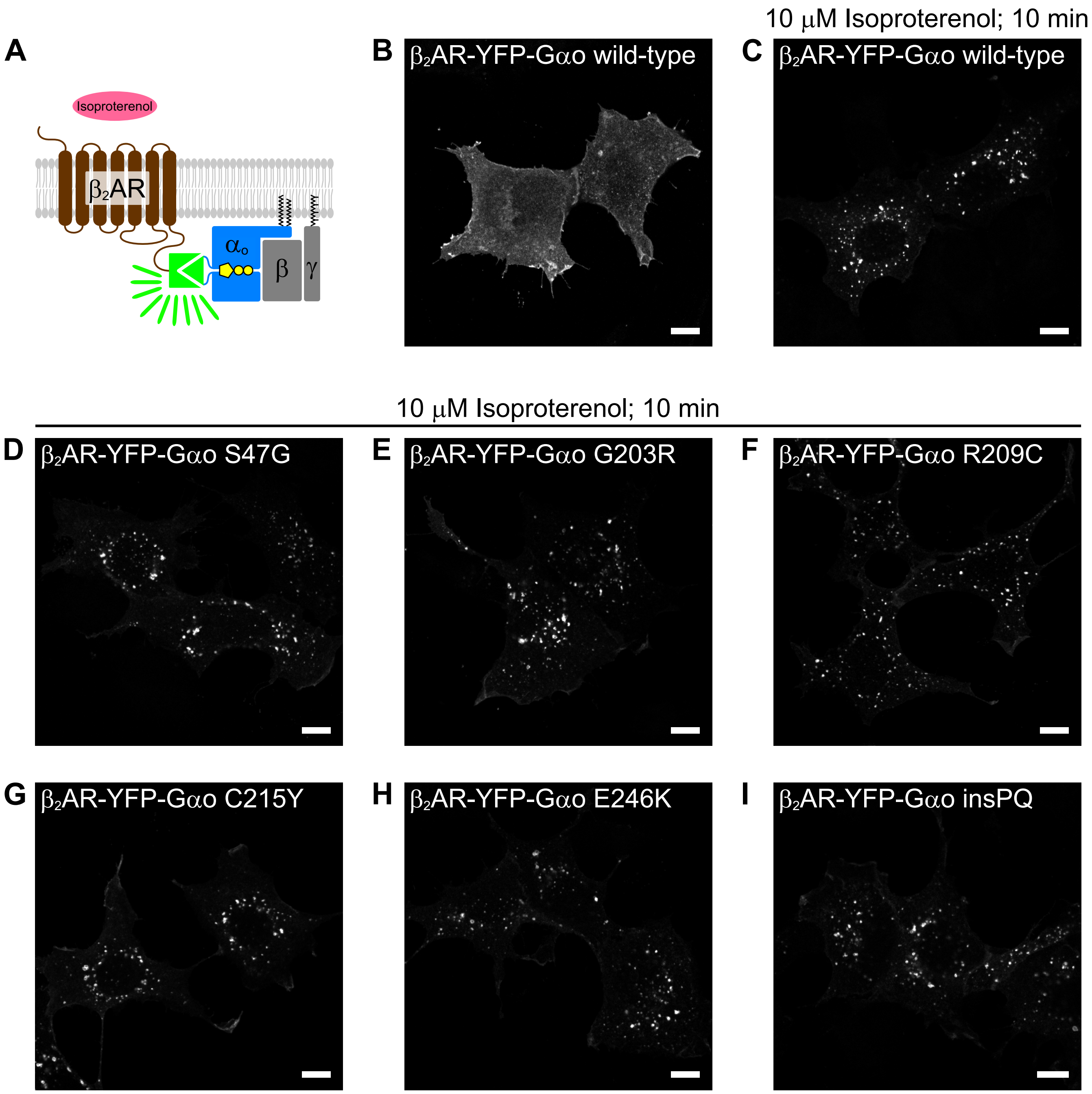

Figure S6. Pathogenic Gαo variants do not affect β_2-adrenoceptor (β_2AR) endocytosis. (**A**) Depiction of the split-YFP assay applied to β_2AR and heterotrimeric Gαoβγ. (**B**,**C**) Representative confocal images of HEK293T cells expressing the β_2AR-YFP-Gαo complex at steady state (**B**) and after 10 min of 10 µM isoproterenol stimulation (**C**). (**D-I**) Confocal images of isoproterenol-stimulated HEK293T cells expressing the β_2AR-YFP-Gαo complex assembled with the indicated Gαo mutants. Scale bars, 10 µm.

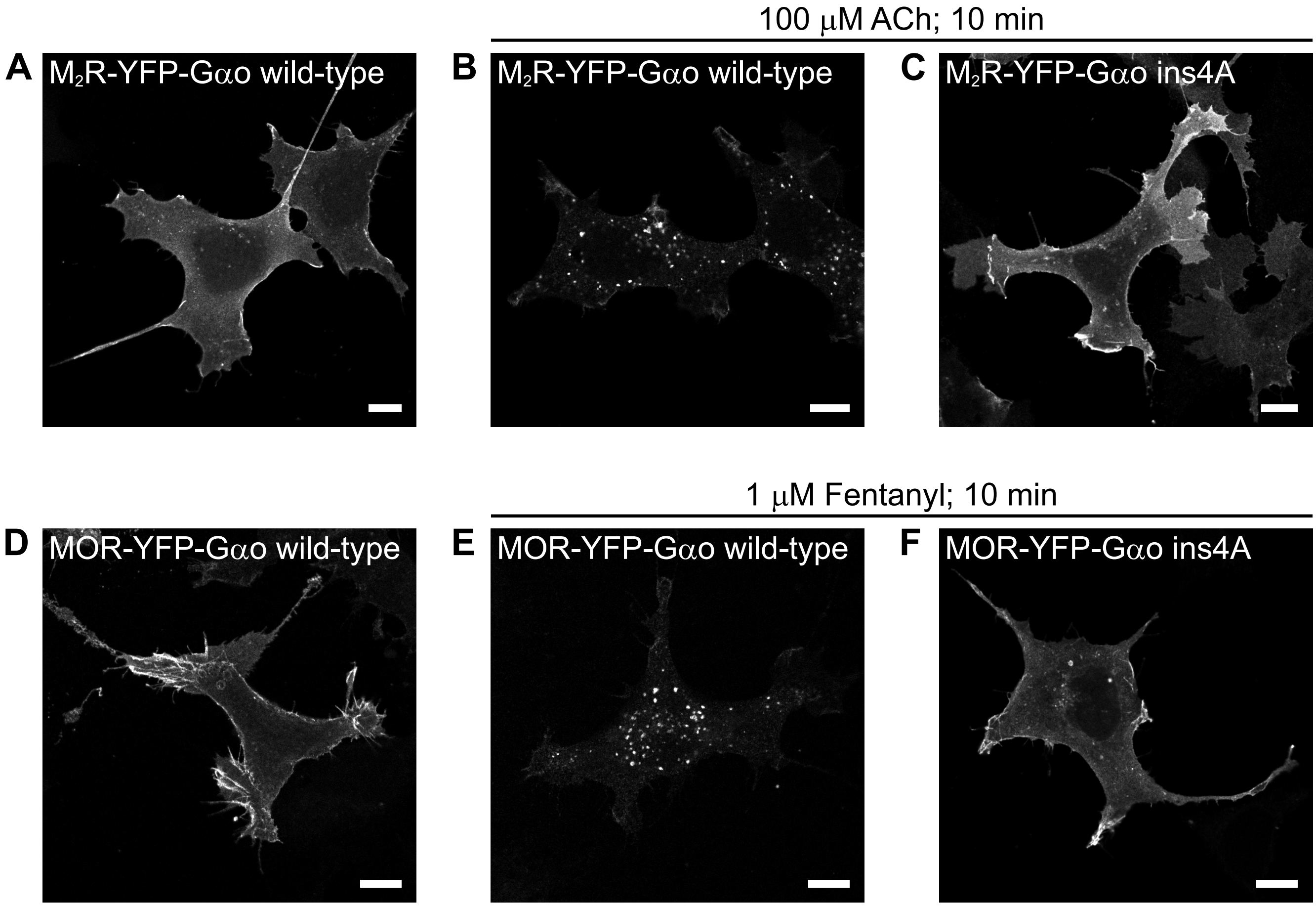

Figure S7. The non-pathogenic Gαo ins4A construct blocks M_2R and MOR endocytosis. (**A**-**F**) Representative confocal images of HEK293T cells expressing the M_2R-YFP-Gαo (**A**-**C**) and MOR-YFP-Gαo (**D**-**F**) complexes assembled with either Gαo wild-type (**A**,**B**,**D**,**E**) and the non-pathogenic ins4A variant (**C**,**F**). Cells were analyzed at steady state (**A**,**D**) and after 10 min stimulation with 100 μM acetylcholine (ACh; **B**,**C**) or 1 μM fentanyl (**E**,**F**). Scale bars, 10 μm.

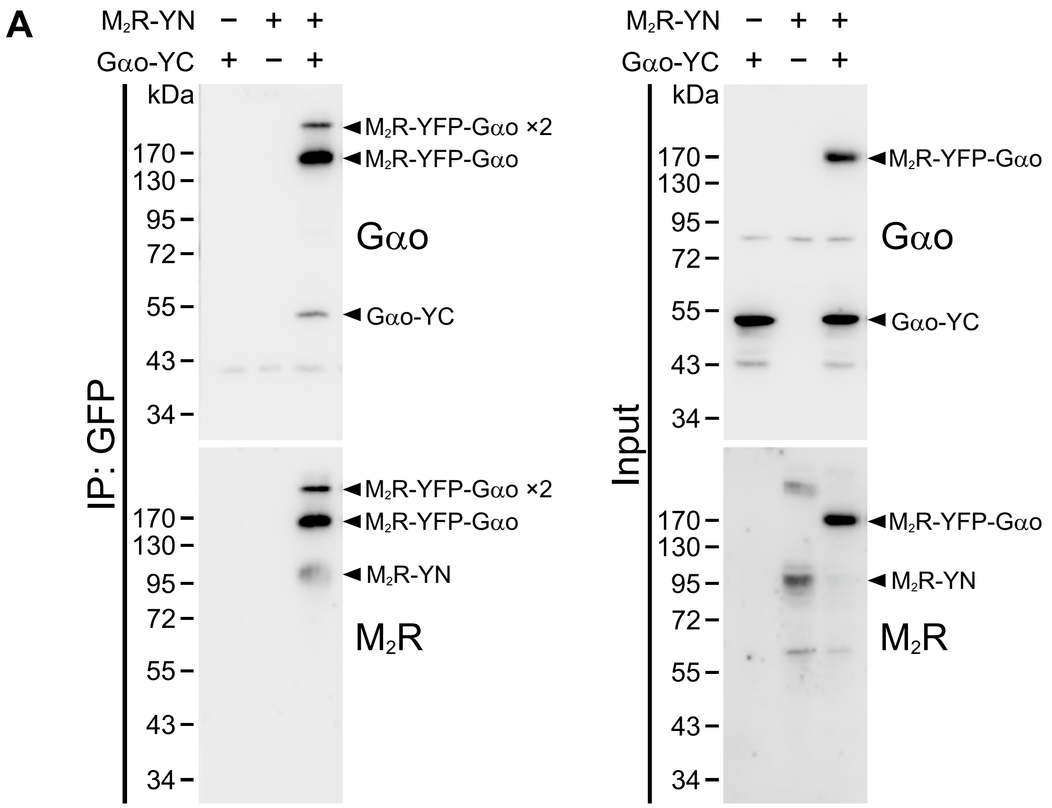

Figure S8. Immunoprecipitation of the M_2R-YFP-Gαo complex. (**A**) HEK293T cells expressing the split-YFP constructs M_2R-YN and Gαo-YC, either individually or in combination as indicated, were subjected to immunoprecipitation (IP) using an anti-GFP nanobody. Western blotting and immunodetection were performed with antibodies against M_2R and Gαo. Arrowheads indicate M_2R-YN, Gαo-YC, and the monomeric and dimeric forms of the M_2R-YFP-Gαo complex.

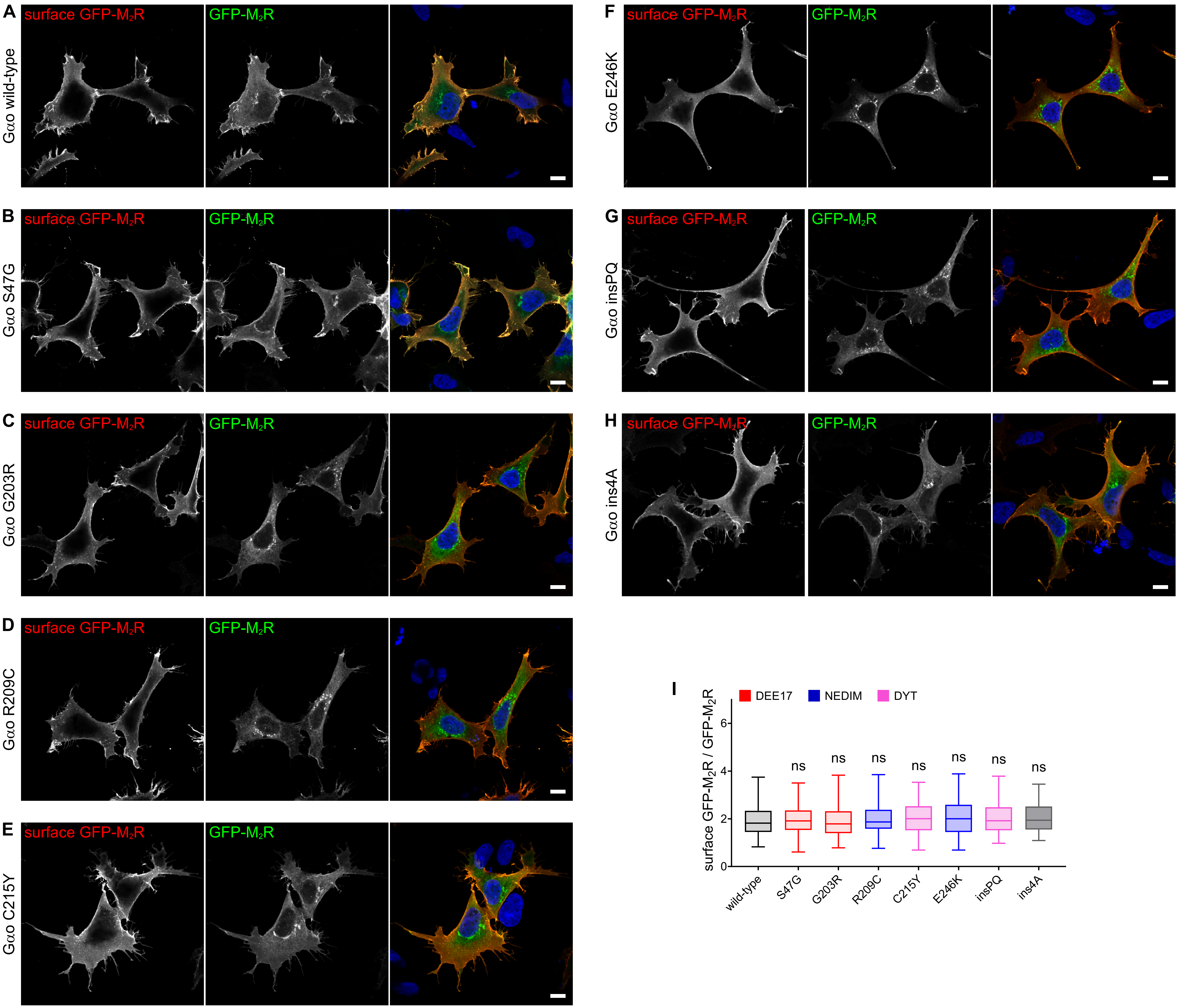

Figure S9. Plasma membrane localization of GFP-M$_2$R in HEK293T cells. (**A-H**) Confocal images of HEK293T cells expressing N-terminally GFP-tagged M$_2$R (GFP-M$_2$R) along with untagged Gαo variants, as indicated. Cells were immunostained with anti-GFP under non-permeabilizing conditions to label extracellular GFP, and counterstained with DAPI to visualize nuclei. Scale bars, 10 µm. (**I**) Quantification of surface M$_2$R levels, measured as the ratio of surface to total GFP-M$_2$R signal. Gαo mutant associations with DEE17, NEDIM and DYT phenotypes are color-coded. Box plots indicate the median (middle line), the 25th and 75th percentiles (box), and the lowest and highest values (whiskers); two independent experiments (wild-type, n = 66; S47G, n = 67; G203R, n = 60; R209C, n = 63; C215Y, n = 69; E246K, n = 64; insPQ, n = 65; ins4A, n = 64). Statistical analysis was done using one-way ANOVA followed by Dunnett's multiple comparisons test; ns: not significant.
